# Macrophage diversity in cancer dissemination and metastasis

**DOI:** 10.1038/s41423-024-01216-z

**Published:** 2024-10-14

**Authors:** Alberto Mantovani, Federica Marchesi, Diletta Di Mitri, Cecilia Garlanda

**Affiliations:** 1 IRCCS Humanitas Research Hospital, Rozzano (Milan), Italy; 2 Department of Biomedical Sciences, Humanitas University, Pieve Emanuele (Milan), Italy; 3 William Harvey Research Institute, Queen Mary University, London, UK; 4 Department Medical Biotechnology and Translational Medicine, Università degli Studi di Milano, Milan, Italy

**Keywords:** Liver, Brain, Bone, Cancer microenvironment, Tumour immunology

## Abstract

Invasion and metastasis are hallmarks of cancer. In addition to the well-recognized hematogenous and lymphatic pathways of metastasis, cancer cell dissemination can occur via the transcoelomic and perineural routes, which are typical of ovarian and pancreatic cancer, respectively. Macrophages are a universal major component of the tumor microenvironment and, in established tumors, promote growth and dissemination to secondary sites. Here, we review the role of tumor-associated macrophages (TAMs) in cancer cell dissemination and metastasis, emphasizing the diversity of myeloid cells in different tissue contexts (lungs, liver, brain, bone, peritoneal cavity, nerves). The generally used models of lung metastasis fail to capture the diversity of pathways and tissue microenvironments. A better understanding of TAM diversity in different tissue contexts may pave the way for tailored diagnostic and therapeutic approaches.

## Introduction

Macrophages play essential physiological roles in ontogenesis and organ function. Moreover, mononuclear phagocytes are central players in chronic inflammation and tissue repair [1–4]. Inflammation is a driver of carcinogenesis in mice and humans [5–8], and myeloid cells are a universal component of the tumor microenvironment (TME) [9–16].

Tumor-associated macrophages (TAMs) can act as double-edged swords [1]. Classically activated macrophages (e.g., by interferon-γ, IFNγ) can kill tumor cells or elicit destructive tumor responses. These M1-like cells are likely to contribute to cancer cell disposal in the early steps of carcinogenesis [17] and to respond to checkpoint blockade immunotherapy [12, 18]. Conversely, in established, metastatic malignant tumors, the net impact of the diversity of TAM clusters in the TME is to promote cancer progression and contribute to resistance to checkpoint blockade immunotherapy [11, 12, 19]. Single-cell analysis and topological localization of macrophages in relation to cancer cells and other immunocompetent cells have added a new dimension of complexity to the current understanding of the TME and the role of TAMs (e.g., [20–35]).

Invasion, dissemination, and metastasis are hallmarks of the malignant behavior of tumors and represent major clinical challenges. Metastasis at different anatomical sites, such as the brain, liver, and bone, poses specific challenges to cytoreductive therapies and immunotherapy. Here, we review the role of mononuclear phagocytes in dissemination and metastasis via the classic hematogenous and lymphatic routes, as well as via the all too frequently forgotten transcoelomic and perineural routes. The emphasis will be on the diversity of TAMs in the distinct ecological niches of different organs and on their clinical implications.

## Pathways of metastasis and the role of TAMs

**Fig. 1:**
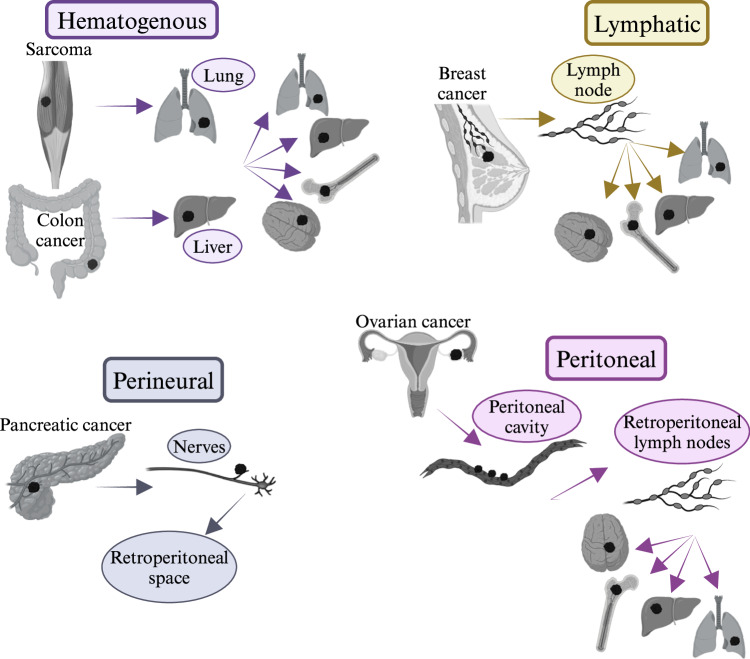
Overview of pathways involved in cancer cell dissemination and metastasis. Cancers of different origins can use different pathways for dissemination: hematogenous, lymphatic, perineural, and transcoelomic. *Created with Biorender.com*

Figure 1 provides an overview of the routes of cancer cell dissemination and metastasis. The hematogenous and lymphatic routes of metastasis are most widely utilized by tumors of different origins, and lymph node involvement is a cornerstone of clinical staging and dictates therapeutic approaches, as exemplified in breast cancer. Podoplanin-expressing TAMs promote lymphangiogenesis, lymphatic vessel invasion, and metastasis [36]. The occurrence of perineural invasion (PNI) and transcoelomic spread through the peritoneal cavity is underestimated, and these pathways of cancer cell dissemination are frequently overlooked. Ovarian cancer and gastrointestinal tumors spread through the peritoneal cavity, interact with lining mesothelial cells, and form secondary neoplastic nests. PNI occurs in many tumors (e.g., colon and breast) but is a major clinical challenge in pancreatic ductal adenocarcinoma.

The tumor-promoting functions of TAMs have been summarized in previous reviews, which provide the framework of the present study (e.g., [9–16]). Schematically, macrophage-mediated tumor promotion includes the following: provision of growth factors; promotion of angiogenesis; suppression and inappropriate skewing of adaptive and innate lymphoid cell-mediated immunity. While these are general mechanisms involved in the immunobiology of TAMs, there is evidence that the tissue context plays a role in shaping the TME, including the phenotype of myeloid cells (e.g., [37–39]). The following sections discuss available information on the role of mononuclear phagocytes in metastasis, with emphasis on the different pathways of dissemination and on the different tissue contextures where seeding and growth occur.

## Metastatic organ niches

### Lung

In patients and preclinical models, the lung is one of the most common sites of cancer metastasis. Dissemination from the primary lesion occurs via hematogenous or lymphatic routes or direct invasion. The cancers that most commonly metastasize to the lung parenchyma are breast, lung, and colorectal cancer uterine leiomyosarcoma and head/neck squamous cell carcinomas, whereas colorectal, renal, and lung cancer, and lymphomas spread to the endobronchial tree.

**Fig. 2:**
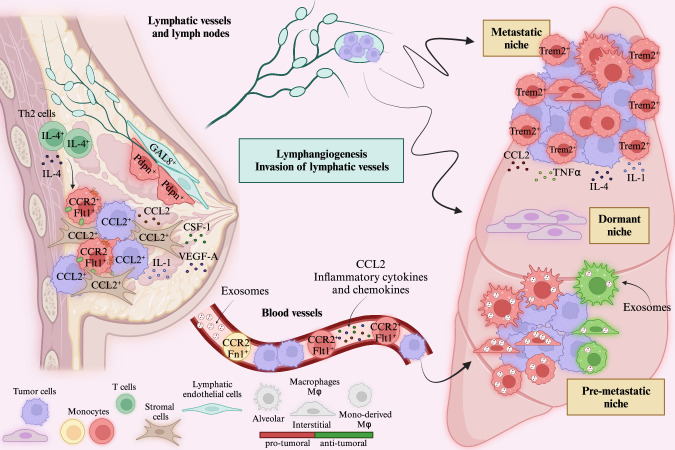
Macrophages are key components of lung metastases. Cytokines, chemokines, and exosomes driving TAM features and prometastatic functions in the primary tumor, premetastatic niche, and metastatic niche are depicted. TAM heterogeneity in terms of origin, transcriptional signatures, localization, and function is illustrated with different colors. TAMs contribute to lymphangiogenesis and invasion of lymphatic vessels and promote tumor cell dissemination through the lymphatic system and hematogenous route. Pdpn podoplanin, Fn1 fibronectin 1. *Created with Biorender.com*

Figure 2 summarizes selected key aspects and molecular players in the pathogenesis of lung metastasis. Seminal preclinical studies on breast cancer metastasis have shown that the metastatic seeding and persistent growth of tumor cells are dependent on macrophages [40, 41]. Similarly, in primary breast tumors and lung metastases, macrophages are among the most abundant immune cells found in the microenvironment, and their frequency positively correlates with reduced overall survival in patients [42]. In these mouse preclinical models, metastases were artificially induced by i.v. injection of Polyoma Middle T-induced tumor cells; however, the relevance of macrophages was confirmed in different spontaneous metastatic breast cancer models. The relevance of macrophages in promoting breast cancer lung metastasis was investigated via a genetic mouse model of macrophage deficiency caused by deficiency of colony stimulating factor-1 (CSF-1), the major macrophage growth factor, and then confirmed by depleting macrophages with liposomes encapsulated with clodronate, even after metastatic growth was established [41].

The mechanisms of metastasis promotion by macrophages include their ability to enhance tumor cell motility, invasion, and intravasation at the primary tumor site; efficient tumor cell extravasation and angiogenesis [11, 12]; and metastasis progression through the promotion of cancer cell extravasation, T-cell suppression, and chemoresistance [43]. Generated by breast tumor cells and stromal cells, CCL2 is the major driver chemokine that recruits CCR2+ inflammatory monocytes and differentiated macrophages during metastasis and activates a chemokine cascade in these cells, which includes the CCL3‒CCR1 axis, further amplifying the process and effectively promoting lung micrometastasis [44, 45]. Initial transcriptional and functional studies revealed that metastasis-associated macrophages are distinct from primary tumor TAMs and express markers associated with inflammatory monocytes and macrophages and the chemokine receptor CCR2 [44, 46]. Lung metastasis-associated macrophages promote survival signals in breast cancer cells (BCCs) by binding to VCAM-1 on cancer cells [46].

Of particular interest is the contribution of myeloid cells and macrophages in particular, in creating the premetastatic niche in which circulating tumor cells settle and grow. Premetastatic niches are preferred metastatic sites in target organs where owing to a preconditioned microenvironment, metastatic tumor cells home, under the control of primary tumor-derived growth factors, chemotactic and inflammatory mediators, as well as tumor-derived exosomes, and thrive [47–49]. The contribution of primary tumor-derived exosomes in instructing lung macrophages has been shown in different preclinical cancer models. The engulfment of tumor-derived microparticles induced transcriptional, phenotypical, and metabolic reprogramming of macrophages in the early metastatic lung in an mTOR-dependent manner, resulting in spatiotemporal metabolic changes driving a functional shift from an antitumor to a protumor state [50]. Similarly, tumor cell-derived exosomes induce an immunosuppressive phenotype in tissue-resident macrophages through glycolytic-dominant metabolic reprogramming, which is associated with increased PD-L1 expression and T-cell suppression [51]. The role of myeloid cells in premetastatic niche formation was demonstrated by the finding that tumor-derived granulocyte colony-stimulating factor (G-CSF) was sufficient to mimic the premetastatic lung microenvironment and promote metastasis in mouse models [52]. In addition, intravascular tissue factor-mediated clot formation in the lungs is associated with the recruitment of macrophages to the premetastatic niche [53]. Macrophages recruited in the premetastatic niche are sources of cytokines, such as IL-1, which promote the recruitment and seeding of metastatic cancer cells at niche sites [1], as well as prometastatic neutrophils [54]. Early on, IL-1 was shown to promote metastasis [55, 56].

Using mouse models of spontaneous metastasis, it has been shown that macrophages of the primary tumor are involved in educating tumor cells for retention, extravasation, and survival in the lung premetastatic niche, as well as the acquisition of stemness and dormancy features [57]. IL-4 is an upstream driver of macrophage-dependent prometastatic functions [58, 59]. IL-4 induces the expression of CCR1, CCL2, and CCL3, which drive monocyte and macrophage recruitment and function. In addition, IL-4 induces the expression of VEGF receptor 1/Flt1, which regulates inflammatory response genes, including colony-stimulating factor 1 (CSF1), a master regulator of macrophage functions [44, 60], in response to VEGFA and increases vascular permeability and tumor cell intravasation [61].

In preclinical lung metastases of the MMTV-PyMT mammary tumor model, combined treatment with chemotherapy or anti-PD-1 therapy with epigenetic reeducation of macrophages with histone deacetylase (HDAC) inhibitors resulted in macrophage reprogramming into proinflammatory and immunostimulatory effectors and increased antitumor effects [62]. Adjuvant epigenetic therapy alone was sufficient to reduce metastatic spread via the inhibition of myeloid cell recruitment to premetastatic niches [63]. Similarly, in osteosarcoma lung metastasis, anti-PD-1 therapy reduces the number of metastases due to macrophage reprogramming into activated tumor M1-like macrophages with a reduction in the number of M2-like macrophages [64]. Re-education of TAMs in highly metastatic mouse models of melanoma and breast and lung cancer has also been performed via immune-metabolic strategies. In particular, inhibition of the enzyme glutamine synthase, which generates glutamine from glutamate, is associated with reprogramming of TAMs into antitumor effectors, resulting in reduced metastasis formation, angiogenesis, and immunosuppression [65]. Effective control of lung metastasis has also been observed in models of sarcoma upon macrophage reprogramming through the combination of TLR agonists, such as poly(I:C) and resiquimod (R848) [66], complement anaphylatoxin inhibition [67], or genetic engineering of IL-12 [68]. In these metastasis models, macrophage reprogramming was associated with effective T-cell-dependent control of tumor growth and metastasis spread, and in the case of C3aR inhibition by the antagonist SB 290157 trifluoroacetate salt, a better response to immunotherapy with anti-PD-1 was observed [67]. In the same model, the macrophage tetraspan MS4A4A, which is specifically expressed in macrophages during differentiation and polarization, is essential for dectin-1-mediated activation of macrophages and natural killer (NK) cell-mediated sarcoma lung metastasis control [69].

Chemotherapy promotes metastasis by increasing the density of perivascular macrophages, which promote tumor revascularization and relapse in both mouse and human tumors [11, 70]. The mechanisms responsible for this pathway include chemotherapy-induced reactive myelopoiesis biased toward monocyte development, increased CCL2 production, and the upregulation of coagulation factor X (FX) in lung interstitial macrophages. Thus, chemotherapy may favor metastasis by promoting the recruitment of monocytes/macrophages and the interplay between coagulation and inflammation in the lungs [71].

Alveolar macrophages are the most abundant population of embryonically derived tissue-resident macrophages in the lung. They are responsible for tuned inflammatory responses and maintenance of metabolic homeostasis under normal conditions and act as a first line of defense in lung infections. Whereas alveolar macrophages are generally self-renewing cells, interstitial macrophages that reside in proximity to blood vessels, nerves, and airways are derived from circulating monocytes and acquire specific features dependent on tissue imprinting [72, 73]. Both tissue-resident macrophages and bone marrow-derived monocytes recruited and differentiated into macrophages at the metastatic site have protumor functions, promoting the survival, migration, and growth of metastatic cells, with differences depending on the experimental model and the metastatic site.

Several efforts have been made in preclinical studies to dissect the heterogeneity and function of specific macrophage subsets in the metastatic process in the lung, with the aim of developing targeted immunotherapeutic approaches that preserve macrophage-mediated antitumor control. Initial studies have shown that bone marrow-derived mature macrophages recruited to the lung after metastatic cell seeding promote their subsequent establishment and growth, whereas yolk sac-derived tissue-resident macrophages, which resist depletion through CSF-1 deficiency, are not involved [41]. In contrast, in a metastatic/orthotopic lung cancer model induced by i.v. injection of cancer cells, both tissue-resident interstitial macrophages, and CCR2-dependent recruited macrophages contributed to the pool of TAMs. Resident macrophages are associated with tumor cell growth in vivo, whereas monocyte-derived TAMs are associated with the support of tumor spread, and after chemotherapy, phagocytosis-mediated tumor clearance occurs [74]. The origin and dynamics of monocyte-derived macrophages and self-maintained tissue-resident macrophages were further investigated in a mouse model of lung adenocarcinoma via a rigorous genetic approach combined with single-cell RNA sequencing (scRNA-seq) and lineage tracing. This study shows that TIL populations differ in origin and have distinct temporal and spatial distributions in the TME. Tissue-resident macrophages are the main source of TAMs and provide a nurturing niche in the very early phases of progression by promoting epithelial–mesenchymal transition and invasiveness in tumor cells and inducing a potent regulatory T-cell response that prevents effective antitumor adaptive immunity. Later, monocyte-derived macrophages are recruited to the tumor site and promote neoplastic development [75]. Resident macrophages, particularly alveolar macrophages, also contribute to the formation of the premetastatic niche by promoting immunosuppression through the inhibition of tumoricidal T helper 1 cell function as well as the maturation of DCs [76].

Transcriptional analysis via scRNA-seq of macrophages from lung metastases of mammary tumors revealed that macrophage subsets are heterogeneous and include populations associated with antigen presentation, self-renewal, immunosuppression, protumorigenic inflammation, myeloid-cell recruitment, and lipid metabolism. In particular, a cluster of lipid-associated macrophages expressing markers of alveolar macrophages, similar to atherosclerosis-associated foamy macrophages, was expanded in metastasis-bearing lungs. This subset was enriched for genes defining pathways related to lipid metabolism, extracellular matrix remodeling, and immunosuppression [77], including lipid-laden macrophages reported within the metastasis of colorectal tumors [20]. Differential transcriptional features of metastasis-associated macrophages have been confirmed by spatial and longitudinal analyses at the single-cell level of the breast tumor metastatic environment within the lung [78]. In particular, enrichment of immunosuppressive TREM2+ macrophages was detected at the invasive margin of the metastasis, both in mouse models and in human samples [78]. Extracellular matrix remodeling is a common feature of lung metastatic macrophages. In spontaneously metastatic models of breast cancer, host-derived osteopontin (OPN, Spp-1), a matricellular protein particularly produced by the monocytic subset of myeloid-derived suppressor cells (MDSCs), renders the metastatic site more immunosuppressive [79].

In contrast with these studies, nonclassical “patrolling” monocytes, which are enriched in the microvasculature of the lung in a CX3CR1-dependent manner, interact with metastasizing tumor cells, scavenge tumor material from the lung vasculature, and promote NK cell recruitment and activation. Through these mechanisms, “patrolling” monocytes reduce tumor metastasis in multiple mouse metastatic tumor models [80].

As discussed above, CCL2 has long been associated with TAM recruitment [81]. A recent study shed new light on the molecular mechanisms responsible for CCL2 production by lung adenocarcinoma (LUAD) [82]. The long noncoding RNA (lncRNA) metastasis-associated lung adenocarcinoma transcript-1 (MALAT1) is correlated with survival and metastasis in several human tumors, including LUAD. In the present study, MALAT1 overexpression in cancer cells promoted metastasis in a noncell autonomous way. MALAT1 increases global chromatin accessibility and CCL2 production, driving the recruitment of protumorigenic TAMs. Although CCL2 here and in previous studies has long been known to contribute to TAM recruitment, in many tumors, clinical trials targeting this chemokine have failed [12]. This historical failure may reflect the multiplicity of pathways driving TAM accumulation.

### Liver

The liver is characterized by unique features in terms of the organization and functions of immunocompetent cells. In general, immunity in the liver is, skewed toward tolerance in many respects, as shown by transplantation and autoimmunity [83–87]. However, the liver represents a first line of innate resistance and is inhabited by a large population of innate lymphoid cells (ILCs), with Kupffer cells serving as a first line of resistance to pathogens entering via the portal blood (e.g., [88]). These peculiarities impact on the role of macrophages in hepatic metastasis.

The liver is a key organ target for tumor metastasis, particularly in colorectal and pancreatic cancer, and macrophages contribute to many aspects of the seeding and organization of the hepatic metastatic niche [89]. The protumor functions of macrophages, such as stimulating angiogenesis, cancer cell invasion into vessels, and metastatic niche formation, facilitate the seeding of cancer cells in the liver. Liver macrophages produce hepatic growth factor that binds to c-Met expressed by tumor cells, promoting their extravasation within the liver [90].

Hepatic macrophages include both Kupffer cells, which are self-renewing and long-lived, and monocyte-derived macrophages, which are rapidly recruited from the circulation into the injured liver [66, 91]. More recently, more specific cluster populations have been described, and the potential of multidimensional analyses is progressively increasing in number.

**Fig. 3:**
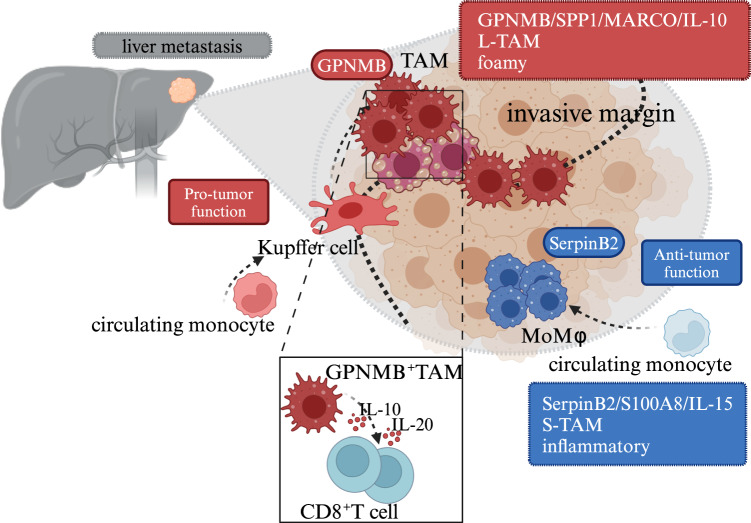
Macrophages in liver metastasis. Distinct populations of macrophages are depicted: MoMϕσ, recently derived from recruited monocytes, express early inflammatory markers (SERPINB2, S100A8), are morphologically small (S-TAMs), and are localized in the peritumoral region. TAMs, on the other hand, overlap more with Kupffer cells, express GPNMB, SPP1, and MARCO, are morphologically large (L-TAMs), are foamy, and are localized in the invasive margin and tumor core. *Created with Biorender.com*

The metastatic liver of colorectal (CRC) cancer patients has been the subject of several studies aimed at dissecting macrophage diversity and involvement in metastatic disease [83, 92–95]. Figure 3 shows selected key populations of macrophages in liver metastases. In general, early activated macrophages, which were recently derived from recruited monocytes, are more frequently associated with antitumor functions, possibly because of limited exposure to the tumor microenvironment [92] (Fig. 3). These cells express early inflammatory markers, such as *SERPINB2* and *S100A8*. TAM signatures, on the other hand, overlap more with resident macrophages than with macrophages recently differentiated from inflammatory monocytes [21]. The key genes expressed by TAMs in CRC liver metastasis are *SPP1*, *TREM2*, *GPNMB*, and *MARCO*. The transcriptomes of these TAM populations are very similar to those of macrophages found in pathological liver conditions, such as lipid-associated macrophages (LAMs) found in steatotic livers and scar-associated macrophages described in liver fibrosis. A comparison of macrophages populating the liver metastatic niche with those found in the primary tumor revealed the liver-specific presence of SPP1^+^ and CCL18^+^ TAMs [93, 94]. The diversity of TAMs was recently investigated in silico via a number of available datasets in an effort to construct a pancancer atlas of infiltrating mononuclear phagocytes [96]. Although metastases were minimally represented in the datasets, the limited data available indicated that the SPP1-expressing cluster was enriched in liver metastases.

Liver metastases are resistant to immunotherapy and represent a clinical challenge [83]. In mouse models, monocyte-derived macrophages in the liver triggered the apoptosis of CD8+ T cells. These findings were mirrored by data from patients whose liver secondaries presented lower tumor T-cell diversity and function [83]. Thus, liver metastasis exploits liver-specific mechanisms of tolerance.

GPNMB was found to be a marker gene of protumor TAMs and was strongly associated with a poor prognosis [92]. Using mice genetically deficient in GPNMB, it was found that GPNMB promoted growth and metastasis in the lungs and liver [97]. GPNMB has complex functions, including skewing of macrophage function and suppression of T-cell immunity. GPNMB has emerged as a marker gene of protumor TAMs in several single-cell studies. These results suggest that this molecule contributes to TAM-mediated tumor progression.

In murine models of liver metastasis from CRC and primary carcinogenesis, macrophages were found to produce IL-18, which stimulated NK cell antitumor effector functions [38, 98].

TAMs in liver metastases can also be classified according to their morphology into large and small TAMs [20]. The two populations have distinct transcriptional profiles; in particular, large TAMs activate cholesterol metabolism and lipid handling (*NR1H3*, *ABCA1*, *CETP*, *FASN*) and are associated with a worse prognosis for patients with metastatic disease [20]. Distinct macrophage populations differ in topological localization, with protumor populations found in tumor regions, which are exposed to tumor-derived and tissue damage signals, and hypoxia, whereas inflammatory populations accumulate more densely in the invasive margin [21].

Metastasis-associated macrophages (MAMs) have also been described for other tumor types; for example, MAMs are required for pancreatic metastasis to the liver [99, 100]. Mechanistically, MAMs secrete granulin, which induces the activation of stellate cells into myofibroblasts, resulting in a protumorigenic fibrotic environment that sustains pancreatic cell tumor growth in the liver [99]. Moreover, granulin-mediated efferocytosis to clear dead parenchymal cells promoted macrophage reprogramming and liver metastasis [100].

IL-1R8 is a negative regulator of members of the IL-1 receptor family [98] and is highly expressed in selected lymphoid populations, including NK cells. Targeting IL-1R8 unleashes NK cell-mediated effector functions in response to IL-18 in murine and human NK cells [38, 101]. IL-1R8 gene-targeted mice are resistant to primary carcinogenesis in the liver but not in other organs and to CRC metastases. The selectivity of the impact of IL-1R8 inactivation was most likely a reflection of the hepatic tissue contexture, characterized by high numbers of ILCs. Thus, while in general, the liver milieu is conducive to tolerance, the high number of ILCs and NK cells, particularly with resident and recruited macrophages, may provide opportunities for therapeutic targeting.

Re-educating macrophages has been pursued via a variety of approaches [12]. Kerzel et al. [102] developed an original strategy to target Kupffer cells and TAMs in liver metastasis. They took advantage of lentiviral vectors, which are preferentially taken up by liver mononuclear phagocytes, and engineered them using the promoter sequence of the mannose receptor, which is expressed in Kupffer cells, M2-like macrophages, and TAMs. They used this lentiviral strategy to engineer the expression of IFNα in Kupffer cells and TAMs. This strategy results in macrophage activation, T-cell activation, reshaping of the TME, and antitumor activity. The reported lentiviral vector-based strategy may pave the way for the development of novel therapeutic strategies in patients with liver metastasis.

The function of mononuclear phagocytes is subjected to negative regulation by a limited set of diverse molecules [12] that inhibit or skew effector functions (myeloid checkpoints). These include the “don’t eat me signal” SIRPα, Clever-1, TREM2, and more. Kerneur et al. [103] recently reported that butyrophilin (BTN)2A1 is highly expressed in TAMs from different cancers, including colorectal liver metastasis. BTN2A1 was found to be upregulated by M2-like signals and by tumor cell supernatants. An anti-BTN2A1 mAb was found to re-educate normal M2-like macrophages and TAMs to exert antitumor effects and reverse the immunosuppressive effects on T cells. The activity of BTN2A1 is mediated by spleen tyrosine kinase (SYK) and ERK phosphorylation. Inhibition of SYK or ERK phosphorylation abolished M2-like reprogramming upon BTN2A1 engagement.

Thus, the dissection of molecular pathways may provide new tools to address the clinical challenge of liver metastasis.

### Bone

Bone metastases are prevalent in advanced stages of various cancers, including prostate, breast, and lung tumors. Bone is a favored site for metastasis, likely because of its highly vascularized nature, which facilitates the seeding of circulating tumor cells. Bone metastases can manifest as either osteoblastic, characterized by increased bone formation, or osteolytic, marked by enhanced bone destruction, and both processes often coexist within the same metastatic lesions [104]. Metastases to bone disrupt bone homeostasis, leading to significant complications, including pain, bone fragility, anemia, and metabolic alterations [105].

The establishment and growth of metastases at distant sites are thought to depend on the intrinsic features of cancer cells, with specific mutations driving dissemination to particular organs. However, the genetic landscape and type of cancer do not fully explain the mechanisms underlying seeding into bone, and interactions between tumor cells and the host environment are considered crucial in influencing the metastatic process [106–108]. The interplay between cancer cells and the bone microenvironment involves various cellular components, including osteoblasts, osteoclasts, endothelial cells, and immune cells, particularly macrophages. Under physiological conditions, macrophages play a crucial role in maintaining bone homeostasis, as evidenced by the neurological and skeletal diseases observed in patients with CSF1R mutations [109]. The bone harbors different macrophage subsets, including osteal macrophages, also called osteomacs, which are associated with bone surfaces, and resident macrophages within the bone marrow [110, 111]. In metastatic lesions, both bone-resident and monocyte-derived macrophages adopt protumoral functions, facilitating cancer progression [112].

**Fig. 4:**
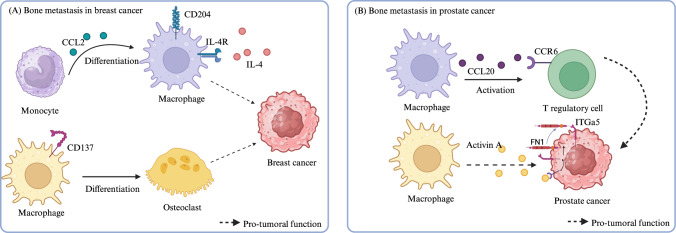
TAMs in bone metastasis. (**A**, upper panel) Monocytes are recruited to the metastatic site through CCL2 and differentiate into CD204 + IL4R+ TAMs that sustain cancer. (**A**, lower panel) CD137 expression on TAMs mediates macrophage migration to the tumor site. CD137+ TAMs differentiate into osteoclasts that support the colonization of cancer cells. (**B**, upper panel) TAMs release CCL20, which engages CCR6 on Tregs, leading to T-cell dysfunction and tumor immune escape. (**B**, lower panel). Activin A released by macrophages activates an FN1-dependent ECM program in tumor cells, resulting in tumor growth. *Created with Biorender.com*

Examination of bone metastases in breast cancer models revealed a subset of macrophages originating from Ly6C+CCR2+ inflammatory monocytes characterized by increased expression of CD204 and IL4R, which have tumor-supportive functions (Fig. 4). Specific deletion of IL4R or blockade of CCR2 attenuated metastatic growth and prolonged survival [112]. Another study on bone lesions in patients with breast cancer revealed that CD137 is expressed by myeloid cells within the bone, promoting monocyte/macrophage migration to the tumor site and differentiation into osteoclasts, thus enhancing metastasis. Administration of a liposomal nanoparticle encapsulating an anti-CD137 antibody significantly inhibited bone metastasis, highlighting the protumoral role of CD137+ macrophages [113].

Notably, the composition of immune cells, including macrophages, differs between primary tumors and metastases. Immunophenotyping of breast cancer patients revealed significant differences in immune cell profiles between primary breast tumors and metastases, with a greater abundance of protumoral macrophages in peripheral lesions. ScRNA-seq and single-cell spatial transcriptomics profiling of breast tumors and matched bone metastases revealed activated macrophages in the stroma of both primary tumors and bone lesions, along with a reduction in lymphoid cells in bone. BMP2 is significantly upregulated in bone tissue, and BMP inhibition in vitro and in mouse models hinders metastatic progression [114].

The heterogeneity of bone macrophages was also observed in prostate cancer lesions. Immunohistochemical analysis revealed diverse macrophage subsets within the tumor mass and pathological woven bone tissue (Fig. 4). Depletion of CD169+ macrophages in an immune-competent mouse model of prostate cancer reduced cancer-induced woven bone formation, highlighting the role of macrophages in bone pathology [115]. ScRNA-seq of bone lesions from prostate cancer patients revealed two monocyte/macrophage subtypes, TIMS (tumor inflammatory monocytes) and TAMS, which are also found in bone metastases of melanoma. A comparison of bone macrophages within solid tumor lesions and matched distant sites revealed an increased proportion of protumoral macrophages in the tumor. Macrophage-expressed CCL20 was identified as an activator of CCR6 in Tregs, leading to the inhibition of cytotoxic T cells. Targeting the CCL20‒CCR6 axis in mouse models resulted in tumor inhibition [116].

Additionally, RNA dataset deconvolution from prostate cancer patients revealed an enrichment of macrophages in bone metastases compared with other organs or primary tumors. Examination of primary tumors and metastatic sites in a model involving the androgen-resistant MycCap cell line with high tropism for bone revealed that TAM-derived Activin A triggers an ECM-related transcription program in cancer cells, leading to enzalutamide resistance. Fibronectin 1 (FN1) activated by activin A sustains SRC-mediated signaling in tumor cells, increasing proliferation [117].

The evidence of an immunosuppressive environment in bone lesions, orchestrated by macrophages, may explain the resistance to therapies at this metastatic site. Notably, bone lesions in cancer patients have been reported to be resistant to immune checkpoint inhibitors, but whether the composition of macrophages dictates resistance still needs to be clarified [118–120]. In light of these findings, targeting bone macrophages appears to be crucial for limiting skeletal complications and underscores potential therapeutic strategies to mitigate the impact of bone tumors and increase the response to therapies.

### Brain

Brain metastases represent up to 90% of all brain malignancies and are associated with significant morbidity and poor prognosis due to the brain’s critical functions and limited regenerative capacity [121]. Several types of primary tumors, including lung cancer, breast cancer, and melanoma, have a high propensity to metastasize to the brain. The preferential invasion of certain tumors rather than others may be due to the distinct features of the brain. Compared with other sites, the CNS is unique because of its location and the requirement for cancer cells to cross the blood‒brain barrier (BBB), which must be disrupted for metastasis to occur. Additionally, the brain TME is dominated by astrocytes and protumorigenic macrophages, with scarce infiltration of lymphocytes [27, 122].

Tissue-resident macrophages, primarily microglia and border-associated macrophages, are abundant in the healthy brain. Microglia, which are identifiable by the expression of P2RY12, TMEM119, and TAL1, are essential for maintaining brain homeostasis. They participate in synaptic pruning, respond to injuries, modulate the endothelium of the BBB, and defend against pathogens. In the context of brain tumors, including metastases, macrophages can also be derived from monocytes recruited from the bloodstream (MDM) and are characterized by markers such as CD49d, FCGR2B, CLEC10A, and CD209 [26, 123]. Both MDMs and resident microglia contribute to tumor progression, influencing tumor growth, invasion, and the response to therapies. High-dimensional approaches have provided significant insights into macrophage characteristics in the central nervous system (CNS) and highlighted differences between primary brain tumors and metastases. Single-cell profiling of tissue samples by mass cytometry and scRNA-seq revealed that the primary tumor niche predominantly features tissue-resident CD49d^low^ microglia, whereas brain metastases are characterized by an accumulation of CD49d^bright^ MDMs [26, 27]. Additionally, brain metastases from primary breast, lung, and melanoma tumors show increased infiltration of T cells with an exhausted phenotype. In brain metastases, TAM populations are more frequently found in neighboring T cells than in primary lesions, indicating possible interactions and T-cell inhibition.

Notably, the TME of brain metastases differs depending on the genetic composition of the tumor (e.g., IDH mutation or wild type) and the type of extracranial tumor of origin. The diverse TMEs and macrophage compositions may explain the divergent responses of primary brain tumors and metastases to therapies. Indeed, studies in mice have shown that phenotypic alterations in TAMs result in antitumor efficacy in glioblastoma [124], whereas TAM depletion prevents brain metastasis outgrowth [125]. Further investigations are needed to shed light on these aspects and enhance our understanding of therapeutic outcomes in these distinct settings [126]. Importantly, CD206+ macrophages are correlated with disease severity in brain malignancies, suggesting that targeting macrophages could be a viable strategy for treating these lesions [27]. Among therapeutic strategies, preclinical studies have demonstrated that inhibitors of CSF1R can achieve antitumor efficacy in brain tumors, although resistance has been observed. Time-course studies have revealed that myeloid cells evolve during tumor treatment, with surviving macrophages becoming dependent on the CSF2-Stat5 pathway. Consequently, combination approaches, including the use of both CSF1 and CSF2 inhibitors, have shown promise in overcoming this resistance [127].

In general, both MDMs and microglia in brain tumors are associated with immunosuppression, suggesting a role in T-cell inhibition [123, 128]. Microglia have also been reported to directly promote extracranial tumor invasion, a process that can be reversed by WNT inhibition [129]. However, certain macrophage subsets may retain their antitumor function. High-dimensional spatial mapping of the brain TME in primary gliomas and metastases through imaging mass cytometry (IMC) identified a unique subset of MPO-positive, neutrophil-like macrophages that appear to increase patient survival [130]. As in other contexts, these findings suggest that selective targeting of macrophages, rather than indiscriminate depletion, should be pursued against brain lesions.

Recently, a novel route of migration for cancer cells through bone to the brain has been described in mice. From the vertebral or calvarial bone marrow, BCCs enter the blood circulation and metastasize to the leptomeninges. Tumor migration is sustained by perivascular macrophages expressing glial-derived neurotrophic factor (GDNF), and disruption of the NCAM-GDNF axis limits metastatic growth in leptomeninges [131].

### Nerves

Nerves are often overlooked components of the TME that contribute to the growth and progression of cancer [132–134], a finding suggested by histopathological evidence of an increased number of nerve fibers within human tumor tissues [135]. Mechanistically, peripheral nerves innervating tumor tissues and exposed to microenvironmental cues modify their transcriptional profile, resulting in structural alterations and the release of neural molecules that can nurture tumor growth [136]. Once invaded by cancer cells, not only does the interaction between neuron cells and tumor cells result in tumor promotion, but nerves can also provide a route for metastasis.

Perineural invasion (PNI), the phenomenon through which cancer cells disseminate along nerves (and possibly spread to distant organs), is a forgotten route of metastasis. Initially, regarded as a passive event, because the connective tissue covering nerves provides minimal resistance to the dissemination of cancer cells, PNI is now considered a well-orchestrated event that comprises bidirectional communication between nerves, cancer cells and immune cells [137]. Over the years, evidence that cancer cells actively migrate along axons has been collected, and both secreted and membrane-bound molecules, including neurotrophic factors (nerve growth factor, GDNF, neurturin, and artemin), chemokines (CXCL13, CX3CL1, and CCL2), neurotransmitters (catecholamines, acetylcholine, and neuropeptides), and adhesion molecules (L1 cell adhesion molecules), have been shown to contribute to PNI [137]. The interaction between nerves and tumor cells is reciprocal: nerves stimulate tumor survival, invasion, and migration, and tumor cells reactivate neural programs, leading to neural sprouting toward cancer cells [138].

In addition to the interaction between nerves and tumor cells, the peripheral nervous system is also known to control the function of immune cells (the so-called neuroimmune axis), including tumor-infiltrating immune cells [139].

Among immune cells, the key players involved in PNI are macrophages, mast cells, and T lymphocytes, which are recruited to invaded nerves via the local release of chemoattractants. Various cell types within the perineurium, such as macrophages, Schwann cells, neurons, and fibroblasts, produce CCL2, a chemoattractant for monocytes and macrophages [140]. Genetic ablation of CCR2 and therefore impaired monocyte recruitment resulted in reduced PNI, suggesting a role for macrophages in this process, whereas pharmacologic inhibition of CSF-1 in a preclinical model of pancreatic cancer significantly reduced the number of endoneurial macrophages and ameliorated the severity of PNI [140]. Mechanistically, endoneurial macrophages support tumor cell growth by secreting high levels of GDNF [137, 140]. Macrophages can also promote neural invasion by cancer cells through the secretion of cathepsin B [141]. Interestingly, pharmacological modulation of the nervous system restrained tumorigenesis by reducing TAM infiltration and TNFα production [142]. In recent work on the relevance of intratumor morphological variants of pancreatic cancer cells, different “biotypes” of cancer cells were found to localize either inside or outside nerves [143]. The differential localization suggested the adaptation of cancer cells to microenvironmental cues, including signals from nerves.

The chemokine axis CX3CR1/CX3CL1 plays a critical role in adhesion between tumor cells and nerves [144]. Pancreatic tumors expressing high levels of CX3CR1 display increased local recurrence, a clinical feature closely linked to the specific tropism of cancer cells for nerves [144].

PNI has been reported as a key pathological feature with clinical relevance and is associated with worse disease in several tumor types, especially pancreatic adenocarcinoma and head and neck, prostate, colorectal, and stomach cancer [137, 144–146]. A perineural inflammatory response characterized by immune cells infiltrating the perineurium of invaded nerves is a typical feature of PNI and is pathogenetic for neuritis [137, 144–146]. Finally, pain, which is frequently associated with neural invasion by cancer cells and in the resulting nerve damage, is a critical aspect of PNI and has an important impact on the quality of life of cancer patients [147, 148]. In a preclinical model of melanoma, Schwann cells released CSF-1, thus sustaining the expansion of macrophages that mediate pain through an oxidative stress mechanism [149].

## Peritoneum and transcoelomic dissemination

**Fig. 5:**
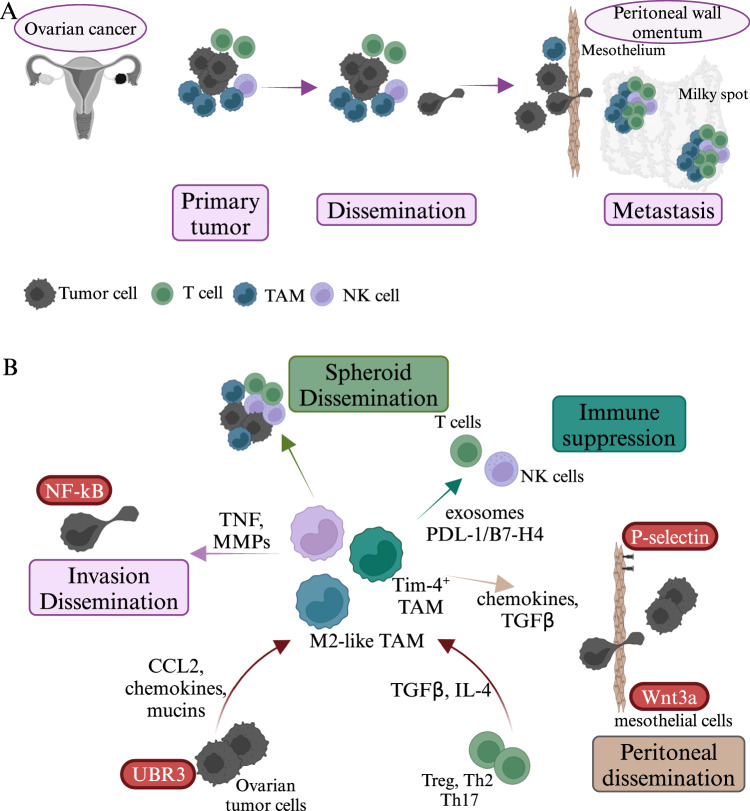
Role of macrophages in transcoelomic dissemination and metastasis. The figure summarizes data in ovarian cancer. **A** Schematic representation of steps in transcoelomic dissemination. **B** Diversity represented as different colors and functions of TAMs in transcoelomic dissemination and peritoneal metastasis formation by ovarian cancer. *Created with Biorender.com*

The peritoneal cavity serves as the major, if not exclusive, dissemination pathway of ovarian cancer, although it is occasionally involved in gastric and colon cancer [150]. Even after hematogenous dissemination, high-grade serous ovarian carcinoma (HGSOC), which accounts for most ovarian cancers, can reach the peritoneum [150]. Dissemination via the transcoelomic route involves tissue-specific mechanisms (Fig. 5A). After leaving the primary tumor, ovarian cancer cells aggregate and form spheroid-like structures in the peritoneal fluid. The omentum is a frequent site of secondary implantation of HGSOCs. Omentum “milky spots” are preferential sites of initial secondary implantation. Milky spots are aggregates of T cells, B cells and macrophages, with interruptions in the mesothelial lining, and serve as niches for metastasis [151].

Ovarian cancer cells, which disseminate as individual elements or aggregates, interact with mesothelial cells, which line the peritoneal cavity. Mesothelial cells play an active role in inflammation and cancer by expressing adhesion molecules (e.g., P selectin, VCAM-1, and ICAM) and by producing and responding to cytokines and chemokines [152, 153].

Macrophages are a major component of the ecological niche of ovarian cancer at the primary tumor site, in the peritoneal fluid, in ascites, and at secondary implantation sites (Fig. 5B). Early on, TAMs from ovarian cancer were the first human phagocyte population in the TME to be shown to promote tumor growth [154–157]. Under homeostatic conditions, inflammation, and cancer, tissue macrophages can originate from embryonic precursors or from circulating monocytes of hematopoietic origin [158]. Ovarian cancer cells and inflammatory cells produce CCL2 and other chemokines that are potent attractants of monocytes in the TME of primary lesions, ascites, and metastases [81, 159]. In a mouse model of ovarian cancer, a subset of tissue-resident macrophages (Tim4+CD163+) has been shown to play a key role in metastatic seeding at these sites [160, 161]. Notably, bone marrow-derived macrophages displace embryo-derived macrophages in serous cavities [162]. It is therefore likely that, in analogy with lung cancer, local tissue-resident mononuclear phagocytes are a source of tumor-promoting TAMs at early stages, with monocyte-derived cells taking the baton later and becoming the dominant population (e.g., [12, 75]).

Myeloid cells infiltrating human neoplasms exhibit substantial diversity, as assessed, for example, by single-cell transcriptomics and IMC [20, 22–32]. Although spatial transcriptomic and/or IMC data are not currently available for ovarian cancer and its metastases, there is evidence of different clusters of TAMs in preclinical models and in clinical HGSOC (e.g., [160, 161, 163–170]) (Fig. 5B). In general, a substantial proportion of TAMs in the TME of HGSOC express molecules such as CD163, CD206, and chemokines, which are associated with an M2-like phenotype [163, 164, 166–168, 170]. The functional orientation of TAM clusters is mediated by complex signals originating from tumor cells and immunocompetent cells (Fig. 5B). Ovarian cancer cells are major drivers of the functional skewing of macrophages [171–174]. Tumor-derived signals include TGFβ, CSF1 and microRNAs delivered via exosomes. Interestingly, ovarian cancer mucins engage the mannose receptor (CD206), which is associated with M2-like polarization, and educate macrophages to perform protumor functions [174]. Similarly, the scavenger receptor (CD163), which is associated with M2-like skewing and is prognostically relevant, was shown to promote progression in a mouse model [175].

Tim4+ TAMs in a mouse model exhibited high oxidative phosphorylation and autophagic adaptation to oxidative stress [169]. In general, metabolism is an essential component of macrophage plasticity and function [176–178]. Metabolic adaptation is a key determinant of the suppression of T-cell responses and the promotion of metastasis [169]. Human ovarian TAMs expressing complement receptors of the Ig superfamily (CR Ig) have similar transcriptional, metabolic, and functional orientations [169]. Thus, macrophages have emerged as friendly companions of cancer cells that metastasize to the peritoneal cavity by affecting all steps of transcoelomic dissemination (Fig. 5).

TAMs obtained from ascites or solid tumors promote tumor cell proliferation [154, 155]. Macrophage-derived growth factors include hepatocyte growth factor-1 (HGF-1), insulin-like growth factor-1 (IGF-1), and TGFβ [179–181]. In groundbreaking sets of experiments, Frances Balkwill and collaborators reported that TNF sets a circuit of ovarian cancer growth in vitro and in vivo (e.g., [182–184]). Macrophages promote invasiveness by activating the NF-κB and JNK pathways [184]. In many tumors, including ovarian cancer, TAMs produce TGFβ and the extracellular proteins tenascin C and fibronectin [185, 186], which have been reported to stimulate migration and metastasis.

Ovarian carcinoma disseminates as single cells and multicellular spheroids [187]. TAMs have been reported to favor the formation of spheroids [188]. Spheroid-like structures may include cells with cancer stem cell properties.

To be implanted throughout the peritoneal cavity, disseminated cancer cells need to interact with mesothelial cells, which express adhesion molecules and are sources and targets of cytokines and chemokines [152, 153]. Ovarian cancer cells engage in complex three-party interactions with macrophages and mesothelial cells [186–189]. M2-like TAMs trigger the upregulation of P-selectin in mesothelial cells via chemokines and enhance cancer cell proliferation, thus favoring seeding at secondary sites [186]. TGFβ induces mesothelial cell chemokine production, especially SDF1/CXCL12, and chemokines such as CCL18 are present in ascites and promote metastasis in the peritoneal cavity [186, 190–192].

The noncanonical Wnt ligand Wnt5a was detected in ovarian ascites. Wnt5a is secreted by mesothelial cells and induces the adhesion of ovarian cancer cells to mesothelial linings and the invasion and formation of secondary lesions [193]. Experiments in gene-targeted mice revealed that in the absence of Wnt5a, high levels of cytotoxic T cells and M1 polarization were observed. These results indicate that a complex bidirectional interaction involving tumor cells, macrophages, and mesothelial cells underlies the secondary localization of ovarian cancer in the peritoneal wall.

Together with Treg cells, myelomonocytic cells are key components of an immunosuppressive microenvironment that characterizes primary and disseminated ovarian cancers. TAMs from ovarian ascites were shown to suppress the function of NK cells and T cells [194]. Interestingly, the suppressive effect of TAMs on NK cells can be rescued by TLR agonists [195, 196]. TAMs from ovarian cancer express PD-L1, which triggers checkpoint blockade and B7-H4 [197, 198]. Moreover, exosomes released by TAMs shift T cells to a Treg phenotype [199].

Interestingly, the E3 ligase UBR3, which is amplified and overexpressed in ovarian cancer, was found to play a nonredundant role in promoting ovarian cancer growth and metastasis [167]. Using gene-targeted mouse and human cell lines, UBR3 was found to drive the production of cytokines and chemokines, TAM recruitment and their immunosuppressive function, and macrophage-enhanced spheroid formation, tumor growth, and metastasis [167].

Consistent with the cellular and molecular networks described above involved in transcoelomatic dissemination, high TAM infiltration has long been strongly associated with an unfavorable prognosis in ovarian cancer, especially when CD163+ cells are considered [92, 170]. TAMs have also been reported to contribute to the resistance of metastatic ovarian cancer to chemotherapy checkpoint blockade immunotherapy [165]. These results raise the issue of diagnostic and therapeutic exploitation of TAMS in this disease (for review, see ref. [12]).

### Concluding remarks

The diversity of tissues where the secondary localization of cancers of the same or different origins occurs represents a clinical challenge. The response to conventional therapies and checkpoint blockade immunotherapy as well as clinical manifestations can pose distinct problems at sites such as the brain, liver, peritoneal cavity and bone, as illustrated by resistance to immunotherapy of hepatic metastases. The evidence summarized here indicates that TAMs in metastatic foci at different organ sites can show substantial differences, presumably reflecting broader peculiarities of the TME and influences of the tissue contexture.

In a recent study in human head and neck squamous cell carcinoma, macrophage plasticity defined by CXCL9 and SPP1 had strong prognostic significance and was associated with a coordinated spatially organized network of TME components [35]. The importance of a simple CXCL9/SPP1 signature was extended to a broad range of human tumors. SPP1 has indeed emerged in a number of studies as a relevant cell cluster marker gene in primary tumors and liver metastasis (e.g., [21, 25, 26, 32, 33]). Assessing the significance of the CXCL9/SPP1 polarity signature in metastasis at different anatomical sites is important.

The emphasis of available information and of the data discussed in this review is on some of the more frequent and clinically relevant sites of metastasis and on myeloid cell properties in these different tissue contexts. However, the bone marrow is frequently seeded early by disseminated tumor cells (e.g., in breast cancer and neuroblastoma), and even if tumor-free, it may affect the function of myeloid cells in the blood and tissues. The bone marrow is a site of secondary neuroblastoma localization. Single-cell transcriptomics and epigenomics have revealed that tumor cells drive the skewing of local monocytes in a protumor direction, mirroring TAMs in solid lesions [200]. In murine and human NSCLC lesions, via scRNA-seq, IL-4 was found to be the main driver of the protumor phenotype of monocyte-derived TAMs [201]. However, the effect of IL-4 occurs at the bone marrow level, with basophils and eosinophils affecting myeloid precursors, which then infiltrate tumors. Therefore, bone marrow precursors may be influenced by cancer cells locally or by systemic signals and may exert remote control over the diversity, phenotype and function of myeloid cells in primary lesions and metastatic foci.

Available information on human metastases from different tumors is still scarce and fragmentary due to obvious difficulties in sample collection. As discussed here, preclinical mouse models of metastasis have been invaluable for establishing fundamental principles, including the role of mononuclear phagocytes in progression and invasion, the formation of a metastatic niche at distant organs, and subsequent metastatic growth. However, the vast majority of studies in preclinical murine models have focused on hematogenous lung metastasis. This represents a major limitation of generally used models, which are not representative of clinically relevant pathways of dissemination. Although fragmentary, the current evidence suggests that diverse pathways and properties of myeloid cells are associated with metastasis at different anatomical sites. Moreover, metastasis to selected organs is associated with resistance to immunotherapy (liver) or poses distinct clinical challenges (e.g., bone).

The results summarized here suggest that one should consider exploiting distinctive features of the organ TME to address resistance, such as IL-1R8, GPNMB, or BTN2A1, for liver metastasis. Macrophage functions are inhibited or skewed by myeloid checkpoints, including the newly discovered BTN2A1 [12, 103]. A systematic analysis of myeloid negative regulators involved in metastasis at different anatomical sites is warranted and may pave the way for novel targeted strategies. Moreover, the liver and transcoelomic metastasis may provide opportunities for the local delivery of selected agents. The use of clinically relevant murine models of metastasis and systematic dissection of the diversity of myeloid cells at metastatic foci in human tumors, owing to their inherent difficulties and limitations, may pave the way for diagnostic and therapeutic approaches focused on TAMs, which are relevant for addressing clinical challenges.

## References

[1] Locati M, Curtale G, Mantovani A. Diversity, mechanisms, and significance of macrophage plasticity. Annu Rev Pathol. 2020;15:123–47. 31530089 10.1146/annurev-pathmechdis-012418-012718 PMC7176483

[2] Wynn TA, Chawla A, Pollard JW. Macrophage biology in development, homeostasis and disease. Nature. 2013;496:445–55. 23619691 10.1038/nature12034 PMC3725458

[3] Mantovani A, Garlanda C. Humoral innate immunity and acute-phase proteins. N. Engl J Med. 2023;388:439–52. 36724330 10.1056/NEJMra2206346 PMC9912245

[4] Park MD, Silvin A, Ginhoux F, Merad M. Macrophages in health and disease. Cell. 2022;185:4259–79. 36368305 10.1016/j.cell.2022.10.007 PMC9908006

[5] Balkwill F, Mantovani A. Inflammation and cancer: back to Virchow? Lancet. 2001;357:539–45. 11229684 10.1016/S0140-6736(00)04046-0

[6] Mantovani A, Allavena P, Sica A, Balkwill F. Cancer-related inflammation. Nature. 2008;454:436–44. 18650914 10.1038/nature07205

[7] Greten FR, Grivennikov SI. Inflammation and cancer: triggers, mechanisms, and consequences. Immunity. 2019;51:27–41. 31315034 10.1016/j.immuni.2019.06.025 PMC6831096

[8] Caronni N, La Terza F, Vittoria FM, Barbiera G, Mezzanzanica L, Cuzzola V, et al. IL-1beta(+) macrophages fuel pathogenic inflammation in pancreatic cancer. Nature. 2023;623:415–22. 37914939 10.1038/s41586-023-06685-2

[9] Fridman WH. The tumor microenvironment: prognostic and theranostic impact. Recent advances and trends. Semin Immunol. 2020;48:101416. 33060021 10.1016/j.smim.2020.101416

[10] Kloosterman DJ, Akkari L. Macrophages at the interface of the coevolving cancer ecosystem. Cell. 2023;186:1627–51. 36924769 10.1016/j.cell.2023.02.020

[11] Mantovani A, Marchesi F, Jaillon S, Garlanda C, Allavena P. Tumor-associated myeloid cells: diversity and therapeutic targeting. Cell Mol Immunol. 2021;18:566–78. 33473192 10.1038/s41423-020-00613-4 PMC8027665

[12] Mantovani A, Allavena P, Marchesi F, Garlanda C. Macrophages as tools and targets in cancer therapy. Nat Rev Drug Discov. 2022;21:799–820. 35974096 10.1038/s41573-022-00520-5 PMC9380983

[13] Joyce JA, Fearon DT. T-cell exclusion, immune privilege, and the tumor microenvironment. Science. 2015;348:74–80. 25838376 10.1126/science.aaa6204

[14] Coussens LM, Zitvogel L, Palucka AK. Neutralizing tumor-promoting chronic inflammation: a magic bullet? Science. 2013;339:286–91. 23329041 10.1126/science.1232227 PMC3591506

[15] De Palma M, Lewis CE. Macrophage regulation of tumor responses to anticancer therapies. Cancer Cell. 2013;23:277–86. 23518347 10.1016/j.ccr.2013.02.013

[16] Pittet MJ, Michielin O, Migliorini D. Clinical relevance of tumor-associated macrophages. Nat Rev Clin Oncol. 2022;19:402–21. 35354979 10.1038/s41571-022-00620-6

[17] Dunn GP, Old LJ, Schreiber RD. The immunobiology of cancer immunosurveillance and immunoediting. Immunity. 2004;21:137–48. 15308095 10.1016/j.immuni.2004.07.017

[18] Kruse B, Buzzai AC, Shridhar N, Braun AD, Gellert S, Knauth K, et al. CD4(+) T-cell-induced inflammatory cell death controls immune-evasive tumors. Nature. 2023;618:1033–40. 37316667 10.1038/s41586-023-06199-x PMC10307640

[19] Morad G, Helmink BA, Sharma P, Wargo JA. Hallmarks of response, resistance, and toxicity to immune checkpoint blockade. Cell. 2022;185:576. 35120665 10.1016/j.cell.2022.01.008

[20] Donadon M, Torzilli G, Cortese N, Soldani C, Di Tommaso L, Franceschini B, et al. Macrophage morphology correlates with single-cell diversity and prognosis in colorectal liver metastasis. J Exp Med. 2020;217:e20191847. 32785653 10.1084/jem.20191847 PMC7596819

[21] Cortese N, Carriero R, Barbagallo M, Putignano AR, Costa G, Giavazzi F, et al. High-resolution analysis of mononuclear phagocytes reveals GPNMB as a prognostic marker in human colorectal liver metastasis. Cancer Immunol Res. 2023;11:405–20. 36652202 10.1158/2326-6066.CIR-22-0462 PMC10070171

[22] Movahedi K, Laoui D, Gysemans C, Baeten M, Stange G, Van den Bossche J, et al. Different tumor microenvironments contain functionally distinct subsets of macrophages derived from Ly6C(high) monocytes. Cancer Res. 2010;70:5728–39. 20570887 10.1158/0008-5472.CAN-09-4672

[23] Lavin Y, Kobayashi S, Leader A, Amir ED, Elefant N, Bigenwald C, et al. Innate immune landscape in early lung adenocarcinoma by paired single-cell analyses. Cell. 2017;169:750–65 e17. 28475900 10.1016/j.cell.2017.04.014 PMC5737939

[24] Lambrechts D, Wauters E, Boeckx B, Aibar S, Nittner D, Burton O, et al. Phenotype molding of stromal cells in the lung tumor microenvironment. Nat Med. 2018;24:1277–89. 29988129 10.1038/s41591-018-0096-5

[25] Zilionis R, Engblom C, Pfirschke C, Savova V, Zemmour D, Saatcioglu HD, et al. Single-cell transcriptomics of human and mouse lung cancers reveals conserved myeloid populations across individuals and species. Immunity. 2019;50:1317–34 e10. 30979687 10.1016/j.immuni.2019.03.009 PMC6620049

[26] Klemm F, Maas RR, Bowman RL, Kornete M, Soukup K, Nassiri S, et al. Interrogation of the microenvironmental landscape in brain tumors reveals disease-specific alterations of immune cells. Cell. 2020;181:1643–60 e17. 32470396 10.1016/j.cell.2020.05.007 PMC8558904

[27] Friebel E, Kapolou K, Unger S, Nunez NG, Utz S, Rushing EJ, et al. Single-cell mapping of human brain cancer reveals tumor-specific instruction of tissue-invading leukocytes. Cell. 2020;181:1626–42 e20. 32470397 10.1016/j.cell.2020.04.055

[28] Azizi E, Carr AJ, Plitas G, Cornish AE, Konopacki C, Prabhakaran S, et al. Single-cell map of diverse immune phenotypes in the breast tumor microenvironment. Cell. 2018;174:1293–308 e36. 29961579 10.1016/j.cell.2018.05.060 PMC6348010

[29] Puram SV, Tirosh I, Parikh AS, Patel AP, Yizhak K, Gillespie S, et al. Single-cell transcriptomic analysis of primary and metastatic tumor ecosystems in head and neck cancer. Cell. 2017;171:1611–24 e24. 29198524 10.1016/j.cell.2017.10.044 PMC5878932

[30] Tirosh I, Izar B, Prakadan SM, Wadsworth MH 2nd, Treacy D, et al. Dissecting the multicellular ecosystem of metastatic melanoma by single-cell RNA-seq. Science. 2016;352:189–96. 27124452 10.1126/science.aad0501 PMC4944528

[31] Chevrier S, Levine JH, Zanotelli VRT, Silina K, Schulz D, Bacac M, et al. An immune atlas of clear cell renal cell carcinoma. Cell. 2017;169:736–49 e18. 28475899 10.1016/j.cell.2017.04.016 PMC5422211

[32] Braun DA, Street K, Burke KP, Cookmeyer DL, Denize T, Pedersen CB, et al. Progressive immune dysfunction with advancing disease stage in renal cell carcinoma. Cancer Cell. 2021;39:632–48 e8. 33711273 10.1016/j.ccell.2021.02.013 PMC8138872

[33] Park MD, Reyes-Torres I, LeBerichel J, Hamon P, LaMarche NM, Hegde S, et al. TREM2 macrophages drive NK cell paucity and dysfunction in lung cancer. Nat Immunol. 2023;24:792–801. 37081148 10.1038/s41590-023-01475-4 PMC11088947

[34] Martinek J, Lin J, Kim KI, Wang VG, Wu TC, Chiorazzi M, et al. Transcriptional profiling of macrophages in situ in metastatic melanoma reveals localization-dependent phenotypes and function. Cell Rep Med. 2022;3:100621. 35584631 10.1016/j.xcrm.2022.100621 PMC9133468

[35] Bill R, Wirapati P, Messemaker M, Roh W, Zitti B, Duval F, et al. CXCL9:SPP1 macrophage polarity identifies a network of cellular programs that control human cancers. Science. 2023;381:515–24. 37535729 10.1126/science.ade2292 PMC10755760

[36] Bieniasz-Krzywiec P, Martin-Perez R, Ehling M, Garcia-Caballero M, Pinioti S, Pretto S, et al. Podoplanin-expressing macrophages promote lymphangiogenesis and lymphoinvasion in breast cancer. Cell Metab. 2019;30:917–36 e10. 31447322 10.1016/j.cmet.2019.07.015 PMC7616630

[37] Ruffell B, Affara NI, Coussens LM. Differential macrophage programming in the tumor microenvironment. Trends Immunol. 2012;33:119–26. 22277903 10.1016/j.it.2011.12.001 PMC3294003

[38] Molgora M, Bonavita E, Ponzetta A, Riva F, Barbagallo M, Jaillon S, et al. IL-1R8 is a checkpoint in NK cells regulating antitumor and anti-viral activity. Nature. 2017;551:110–4. 29072292 10.1038/nature24293 PMC5768243

[39] Zhong J, Xing X, Gao Y, Pei L, Lu C, Sun H, et al. Distinct roles of TREM2 in central nervous system cancers and peripheral cancers. Cancer Cell. 2024;42:968–84 e9. 38788719 10.1016/j.ccell.2024.05.001

[40] Lin EY, Nguyen AV, Russell RG, Pollard JW. Colony-stimulating factor 1 promotes progression of mammary tumors to malignancy. J Exp Med. 2001;193:727–40. 11257139 10.1084/jem.193.6.727 PMC2193412

[41] Qian B, Deng Y, Im JH, Muschel RJ, Zou Y, Li J, et al. A distinct macrophage population mediates metastatic breast cancer cell extravasation, establishment and growth. PLoS ONE. 2009;4:e6562. 19668347 10.1371/journal.pone.0006562 PMC2721818

[42] Medrek C, Ponten F, Jirstrom K, Leandersson K. The presence of tumor associated macrophages in tumor stroma as a prognostic marker for breast cancer patients. BMC Cancer. 2012;12:306. 22824040 10.1186/1471-2407-12-306 PMC3414782

[43] Lin EY, Pollard JW. Tumor-associated macrophages press the angiogenic switch in breast cancer. Cancer Res. 2007;67:5064–6. 17545580 10.1158/0008-5472.CAN-07-0912

[44] Qian BZ, Li J, Zhang H, Kitamura T, Zhang J, Campion LR, et al. CCL2 recruits inflammatory monocytes to facilitate breast-tumor metastasis. Nature. 2011;475:222–5. 21654748 10.1038/nature10138 PMC3208506

[45] Kitamura T, Qian BZ, Soong D, Cassetta L, Noy R, Sugano G, et al. CCL2-induced chemokine cascade promotes breast cancer metastasis by enhancing retention of metastasis-associated macrophages. J Exp Med. 2015;212:1043–59. 26056232 10.1084/jem.20141836 PMC4493415

[46] Lu X, Kang Y. Chemokine (C-C motif) ligand 2 engages CCR2+ stromal cells of monocytic origin to promote breast cancer metastasis to lung and bone. J Biol Chem. 2009;284:29087–96. 19720836 10.1074/jbc.M109.035899 PMC2781454

[47] Hiratsuka S, Watanabe A, Aburatani H, Maru Y. Tumor-mediated upregulation of chemoattractants and recruitment of myeloid cells predetermines lung metastasis. Nat Cell Biol. 2006;8:1369–75. 17128264 10.1038/ncb1507

[48] Kaplan RN, Riba RD, Zacharoulis S, Bramley AH, Vincent L, Costa C, et al. VEGFR1-positive hematopoietic bone marrow progenitors initiate the premetastatic niche. Nature. 2005;438:820–7. 16341007 10.1038/nature04186 PMC2945882

[49] Kim S, Takahashi H, Lin WW, Descargues P, Grivennikov S, Kim Y, et al. Carcinoma-produced factors activate myeloid cells through TLR2 to stimulate metastasis. Nature. 2009;457:102–6. 19122641 10.1038/nature07623 PMC2746432

[50] Kersten K, You R, Liang S, Tharp KM, Pollack J, Weaver VM, et al. Uptake of tumor-derived microparticles induces metabolic reprogramming of macrophages in the early metastatic lung. Cell Rep. 2023;42:112582. 37261951 10.1016/j.celrep.2023.112582 PMC10592447

[51] Morrissey SM, Zhang F, Ding C, Montoya-Durango DE, Hu X, Yang C, et al. Tumor-derived exosomes drive immunosuppressive macrophages in a premetastatic niche through glycolytic dominant metabolic reprogramming. Cell Metab. 2021;33:2040–58 e10. 34559989 10.1016/j.cmet.2021.09.002 PMC8506837

[52] Shojaei F, Wu X, Qu X, Kowanetz M, Yu L, Tan M, et al. G-CSF-initiated myeloid cell mobilization and angiogenesis mediate tumor refractoriness to anti-VEGF therapy in mouse models. Proc Natl Acad Sci USA. 2009;106:6742–7. 19346489 10.1073/pnas.0902280106 PMC2665197

[53] Gil-Bernabe AM, Ferjancic S, Tlalka M, Zhao L, Allen PD, Im JH, et al. Recruitment of monocytes/macrophages by tissue factor-mediated coagulation is essential for metastatic cell survival and premetastatic niche establishment in mice. Blood. 2012;119:3164–75. 22327225 10.1182/blood-2011-08-376426

[54] Wellenstein MD, Coffelt SB, Duits DEM, van Miltenburg MH, Slagter M, de Rink I, et al. Loss of p53 triggers WNT-dependent systemic inflammation to drive breast cancer metastasis. Nature. 2019;572:538–42. 31367040 10.1038/s41586-019-1450-6 PMC6707815

[55] Giavazzi R, Garofalo A, Bani MR, Abbate M, Ghezzi P, Boraschi D, et al. Interleukin 1-induced augmentation of experimental metastases from a human melanoma in nude mice. Cancer Res. 1990;50:4771–5. 2196116

[56] Garlanda C, Mantovani A. Interleukin-1 in tumor progression, therapy, and prevention. Cancer Cell. 2021;39:1023–7. 33989512 10.1016/j.ccell.2021.04.011

[57] Borriello L, Coste A, Traub B, Sharma VP, Karagiannis GS, Lin Y, et al. Primary tumor associated macrophages activate programs of invasion and dormancy in disseminating tumor cells. Nat Commun. 2022;13:626. 35110548 10.1038/s41467-022-28076-3 PMC8811052

[58] DeNardo DG, Barreto JB, Andreu P, Vasquez L, Tawfik D, Kolhatkar N, et al. CD4(+) T cells regulate pulmonary metastasis of mammary carcinomas by enhancing protumor properties of macrophages. Cancer Cell. 2009;16:91–102. 19647220 10.1016/j.ccr.2009.06.018 PMC2778576

[59] Rodriguez-Tirado C, Entenberg D, Li J, Qian BZ, Condeelis JS, Pollard JW. Interleukin 4 controls the pro-tumoral role of macrophages in mammary cancer pulmonary metastasis in mice. Cancers. 2022;14:4336. 36077870 10.3390/cancers14174336 PMC9454655

[60] Qian BZ, Zhang H, Li J, He T, Yeo EJ, Soong DY, et al. FLT1 signaling in metastasis-associated macrophages activates an inflammatory signature that promotes breast cancer metastasis. J Exp Med. 2015;212:1433–48. 26261265 10.1084/jem.20141555 PMC4548055

[61] Harney AS, Arwert EN, Entenberg D, Wang Y, Guo P, Qian BZ, et al. Real-time imaging reveals local, transient vascular permeability, and tumor cell intravasation stimulated by TIE2hi macrophage-derived VEGFA. Cancer Discov. 2015;5:932–43. 26269515 10.1158/2159-8290.CD-15-0012 PMC4560669

[62] Guerriero JL, Sotayo A, Ponichtera HE, Castrillon JA, Pourzia AL, Schad S, et al. Class IIa HDAC inhibition reduces breast tumors and metastases through antitumor macrophages. Nature. 2017;543:428–32. 28273064 10.1038/nature21409 PMC8170529

[63] Lu Z, Zou J, Li S, Topper MJ, Tao Y, Zhang H, et al. Epigenetic therapy inhibits metastases by disrupting premetastatic niches. Nature. 2020;579:284–90. 32103175 10.1038/s41586-020-2054-x PMC8765085

[64] Dhupkar P, Gordon N, Stewart J, Kleinerman ES. Anti-PD-1 therapy redirects macrophages from an M2 to an M1 phenotype inducing regression of OS lung metastases. Cancer Med. 2018;7:2654–64. 29733528 10.1002/cam4.1518 PMC6010882

[65] Menga A, Serra M, Todisco S, Riera-Domingo C, Ammarah U, Ehling M, et al. Glufosinate constrains synchronous and metachronous metastasis by promoting anti-tumor macrophages. EMBO Mol Med. 2020;12:e11210. 32885605 10.15252/emmm.201911210 PMC7539200

[66] Anfray C, Mainini F, Digifico E, Maeda A, Sironi M, Erreni M, et al. Intratumoral combination therapy with poly(I:C) and resiquimod synergistically triggers tumor-associated macrophages for effective systemic antitumoral immunity. J Immunother Cancer. 2021;9:e002408. 10.1136/jitc-2021-002408 PMC8449972 34531246

[67] Magrini E, Di Marco S, Mapelli SN, Perucchini C, Pasqualini F, Donato A, et al. Complement activation promoted by the lectin pathway mediates C3aR-dependent sarcoma progression and immunosuppression. Nat Cancer. 2021;2:218–32. 34505065 10.1038/s43018-021-00173-0 PMC8425276

[68] Kaczanowska S, Beury DW, Gopalan V, Tycko AK, Qin H, Clements ME, et al. Genetically engineered myeloid cells rebalance the core immune suppression program in metastasis. Cell. 2021;184:2033–52 e21. 33765443 10.1016/j.cell.2021.02.048 PMC8344805

[69] Mattiola I, Tomay F, De Pizzol M, Silva-Gomes R, Savino B, Gulic T, et al. The macrophage tetraspan MS4A4A enhances dectin-1-dependent NK cell-mediated resistance to metastasis. Nat Immunol. 2019;20:1012–22. 31263276 10.1038/s41590-019-0417-y PMC7176488

[70] Karagiannis GS, Pastoriza JM, Wang Y, Harney AS, Entenberg D, Pignatelli J, et al. Neoadjuvant chemotherapy induces breast cancer metastasis through a TMEM-mediated mechanism. Sci Transl Med. 2017;9:eaan0026. 28679654 10.1126/scitranslmed.aan0026 PMC5592784

[71] Wu C, Zhong Q, Shrestha R, Wang J, Hu X, Li H, et al. Reactive myelopoiesis and FX-expressing macrophages triggered by chemotherapy promote cancer lung metastasis. JCI Insight. 2023;8:e167499. 36976637 10.1172/jci.insight.167499 PMC10243818

[72] Tan SY, Krasnow MA. Developmental origin of lung macrophage diversity. Development. 2016;143:1318–27. 26952982 10.1242/dev.129122 PMC4852511

[73] Chakarov S, Lim HY, Tan L, Lim SY, See P, Lum J, et al. Two distinct interstitial macrophage populations coexist across tissues in specific subtissular niches. Science. 2019;363:eaau0964. 10.1126/science.aau0964 30872492

[74] Loyher PL, Hamon P, Laviron M, Meghraoui-Kheddar A, Goncalves E, Deng Z, et al. Macrophages of distinct origins contribute to tumor development in the lung. J Exp Med. 2018;215:2536–53. 30201786 10.1084/jem.20180534 PMC6170177

[75] Casanova-Acebes M, Dalla E, Leader AM, LeBerichel J, Nikolic J, Morales BM, et al. Tissue-resident macrophages provide a pro-tumorigenic niche to early NSCLC cells. Nature. 2021;595:578–84. 34135508 10.1038/s41586-021-03651-8 PMC8923521

[76] Sharma SK, Chintala NK, Vadrevu SK, Patel J, Karbowniczek M, Markiewski MM. Pulmonary alveolar macrophages contribute to the premetastatic niche by suppressing antitumor T-cell responses in the lungs. J Immunol. 2015;194:5529–38. 25911761 10.4049/jimmunol.1403215

[77] Huggins DN, LaRue RS, Wang Y, Knutson TP, Xu Y, Williams JW, et al. Characterizing macrophage diversity in metastasis-bearing lungs reveals a lipid-associated macrophage subset. Cancer Res. 2021;81:5284–95. 34389631 10.1158/0008-5472.CAN-21-0101 PMC8530952

[78] Yofe I, Shami T, Cohen N, Landsberger T, Sheban F, Stoler-Barak L, et al. Spatial and temporal mapping of breast cancer lung metastases identify TREM2 macrophages as regulators of the metastatic boundary. Cancer Discov. 2023;13:2610–31. 37756565 10.1158/2159-8290.CD-23-0299 PMC7617931

[79] Sangaletti S, Tripodo C, Sandri S, Torselli I, Vitali C, Ratti C, et al. Osteopontin shapes immunosuppression in the metastatic niche. Cancer Res. 2014;74:4706–19. 25035397 10.1158/0008-5472.CAN-13-3334

[80] Hanna RN, Cekic C, Sag D, Tacke R, Thomas GD, Nowyhed H, et al. Patrolling monocytes control tumor metastasis to the lung. Science. 2015;350:985–90. 26494174 10.1126/science.aac9407 PMC4869713

[81] Bottazzi B, Polentarutti N, Acero R, Balsari A, Boraschi D, Ghezzi P, et al. Regulation of the macrophage content of neoplasms by chemoattractants. Science. 1983;220:210–2. 6828888 10.1126/science.6828888

[82] Martinez-Terroba E, Plasek-Hegde LM, Chiotakakos I, Li V, de Miguel FJ, Robles-Oteiza C, et al. Overexpression of Malat1 drives metastasis through inflammatory reprogramming of the tumor microenvironment. Sci Immunol. 2024;9:eadh5462. 38875320 10.1126/sciimmunol.adh5462 PMC12087577

[83] Yu J, Green MD, Li S, Sun Y, Journey SN, Choi JE, et al. Liver metastasis restrains immunotherapy efficacy via macrophage-mediated T-cell elimination. Nat Med. 2021;27:152–64. 33398162 10.1038/s41591-020-1131-x PMC8095049

[84] Li F, Tian Z. The liver works as a school to educate regulatory immune cells. Cell Mol Immunol. 2013;10:292–302. 23604044 10.1038/cmi.2013.7 PMC4003213

[85] Doherty DG. Immunity, tolerance and autoimmunity in the liver: a comprehensive review. J Autoimmun. 2016;66:60–75. 26358406 10.1016/j.jaut.2015.08.020

[86] Crispe IN. Hepatic T cells and liver tolerance. Nat Rev Immunol. 2003;3:51–62. 12511875 10.1038/nri981

[87] Crispe IN, Dao T, Klugewitz K, Mehal WZ, Metz DP. The liver as a site of T-cell apoptosis: graveyard, or killing field? Immunol Rev. 2000;174:47–62. 10807506 10.1034/j.1600-0528.2002.017412.x

[88] Wong CH, Jenne CN, Petri B, Chrobok NL, Kubes P. Nucleation of platelets with blood-borne pathogens on Kupffer cells precedes other innate immunity and contributes to bacterial clearance. Nat Immunol. 2013;14:785–92. 23770641 10.1038/ni.2631 PMC4972575

[89] Peinado H, Zhang H, Matei IR, Costa-Silva B, Hoshino A, Rodrigues G, et al. Premetastatic niches: organ-specific homes for metastases. Nat Rev Cancer. 2017;17:302–17. 28303905 10.1038/nrc.2017.6

[90] Kitamura T, Kato Y, Brownlie D, Soong DYH, Sugano G, Kippen N, et al. Mammary tumor cells with high metastatic potential are hypersensitive to macrophage-derived HGF. Cancer Immunol Res. 2019;7:2052–64. 31615815 10.1158/2326-6066.CIR-19-0234 PMC6891215

[91] Bleriot C, Ginhoux F. Understanding the heterogeneity of resident liver macrophages. Front Immunol. 2019;10:2694. 31803196 10.3389/fimmu.2019.02694 PMC6877662

[92] Cortese N, Carriero R, Laghi L, Mantovani A, Marchesi F. Prognostic significance of tumor-associated macrophages: past, present and future. Semin Immunol. 2020;48:101408. 32943279 10.1016/j.smim.2020.101408

[93] Wu Y, Yang S, Ma J, Chen Z, Song G, Rao D, et al. Spatiotemporal immune landscape of colorectal cancer liver metastasis at single-cell level. Cancer Discov. 2022;12:134–53. 34417225 10.1158/2159-8290.CD-21-0316

[94] Liu Y, Zhang Q, Xing B, Luo N, Gao R, Yu K, et al. Immune phenotypic linkage between colorectal cancer and liver metastasis. Cancer Cell. 2022;40:424–37 e5. 35303421 10.1016/j.ccell.2022.02.013

[95] Zhang L, Li Z, Skrzypczynska KM, Fang Q, Zhang W, O’Brien SA, et al. Single-cell analyses inform mechanisms of myeloid-targeted therapies in colon cancer. Cell. 2020;181:442–59 e29. 32302573 10.1016/j.cell.2020.03.048

[96] Coulton A, Murai J, Qian D, Thakkar K, Lewis CE, Litchfield K. Using a pancancer atlas to investigate tumor associated macrophages as regulators of immunotherapy response. Nat Commun. 2024;15:5665. 38969631 10.1038/s41467-024-49885-8 PMC11226649

[97] Liguori M, Digifico E, Vacchini A, Avigni R, Colombo FS, Borroni EM, et al. The soluble glycoprotein NMB (GPNMB) produced by macrophages induces cancer stemness and metastasis via CD44 and IL-33. Cell Mol Immunol. 2021;18:711–22. 32728200 10.1038/s41423-020-0501-0 PMC8027814

[98] Mantovani A, Dinarello CA, Molgora M, Garlanda C. Interleukin-1 and related cytokines in the regulation of inflammation and immunity. Immunity. 2019;50:778–95. 30995499 10.1016/j.immuni.2019.03.012 PMC7174020

[99] Nielsen SR, Quaranta V, Linford A, Emeagi P, Rainer C, Santos A, et al. Macrophage-secreted granulin supports pancreatic cancer metastasis by inducing liver fibrosis. Nat Cell Biol. 2016;18:549–60. 27088855 10.1038/ncb3340 PMC4894551

[100] Astuti Y, Raymant M, Quaranta V, Clarke K, Abudula M, Smith O, et al. Author Correction: Efferocytosis reprograms the tumor microenvironment to promote pancreatic cancer liver metastasis. Nat Cancer. 2024;5:808. 38472300 10.1038/s43018-024-00751-y PMC11136648

[101] Landolina N, Mariotti FR, Ingegnere T, Alicata C, Ricci B, Pelosi A, et al. IL-1R8 silencing improves the anti-tumor function of freshly isolated human NK cells. J Immunother Cancer. 2022;10:e003858. 35292515 10.1136/jitc-2021-003858 PMC8928329

[102] Kerzel T, Giacca G, Beretta S, Bresesti C, Notaro M, Scotti GM, et al. In vivo macrophage engineering reshapes the tumor microenvironment leading to eradication of liver metastases. Cancer Cell. 2023;41:1892–910 e10. 37863068 10.1016/j.ccell.2023.09.014

[103] Kerneur C, Foucher E, Guillen Casas J, Colazet M, Le K-S, Fullana M, et al. BTN2A1 targeting reprograms M2-like macrophagesand TAMs via SYK and MAPK signaling. Cell Rep. 2024;43:114773. 10.1016/j.celrep.2024.114773 39325623

[104] Mundy GR. Metastasis to bone: causes, consequences and therapeutic opportunities. Nat Rev Cancer. 2002;2:584–93. 12154351 10.1038/nrc867

[105] Suva LJ, Washam C, Nicholas RW, Griffin RJ. Bone metastasis: mechanisms and therapeutic opportunities. Nat Rev Endocrinol. 2011;7:208–18. 21200394 10.1038/nrendo.2010.227 PMC3134309

[106] Chen F, Han Y, Kang Y. Bone marrow niches in the regulation of bone metastasis. Br J Cancer. 2021;124:1912–20. 33758331 10.1038/s41416-021-01329-6 PMC8184962

[107] Kang Y, Siegel PM, Shu W, Drobnjak M, Kakonen SM, Cordon-Cardo C, et al. A multigenic program mediating breast cancer metastasis to bone. Cancer Cell. 2003;3:537–49. 12842083 10.1016/s1535-6108(03)00132-6

[108] Masetti M, Carriero R, Portale F, Marelli G, Morina N, Pandini M, et al. Lipid-loaded tumor-associated macrophages sustain tumor growth and invasiveness in prostate cancer. J Exp Med. 2022;219:e20210564. 34919143 10.1084/jem.20210564 PMC8932635

[109] Lazarov T, Juarez-Carreno S, Cox N, Geissmann F. Publisher Correction: Physiology and diseases of tissue-resident macrophages. Nature. 2023;619:E51. 37400553 10.1038/s41586-023-06386-w

[110] Mass E, Nimmerjahn F, Kierdorf K, Schlitzer A. Tissue-specific macrophages: how they develop and choreograph tissue biology. Nat Rev Immunol. 2023;23:563–79. 36922638 10.1038/s41577-023-00848-y PMC10017071

[111] Davies LC, Jenkins SJ, Allen JE, Taylor PR. Tissue-resident macrophages. Nat Immunol. 2013;14:986–95. 24048120 10.1038/ni.2705 PMC4045180

[112] Ma RY, Zhang H, Li XF, Zhang CB, Selli C, Tagliavini G, et al. Monocyte-derived macrophages promote breast cancer bone metastasis outgrowth. J Exp Med. 2020;217:20191820. 10.1084/jem.20191820 PMC7596825 32780802

[113] Zhu L, Narloch JL, Onkar S, Joy M, Broadwater G, Luedke C, et al. Metastatic breast cancers have reduced immune cell recruitment but harbor increased macrophages relative to their matched primary tumors. J Immunother Cancer. 2019;7:265. 31627744 10.1186/s40425-019-0755-1 PMC6798422

[114] Ihle CL, Straign DM, Canari JA, Torkko KC, Zolman KL, Smith EE, et al. Unique macrophage phenotypes activated by BMP signaling in breast cancer bone metastases. JCI Insight. 2024;9:e168517. 38193534 10.1172/jci.insight.168517 PMC10906463

[115] Wu AC, He Y, Broomfield A, Paatan NJ, Harrington BS, Tseng HW, et al. CD169(+) macrophages mediate pathological formation of woven bone in skeletal lesions of prostate cancer. J Pathol. 2016;239:218–30. 27174786 10.1002/path.4718

[116] Kfoury Y, Baryawno N, Severe N, Mei S, Gustafsson K, Hirz T, et al. Human prostate cancer bone metastases have an actionable immunosuppressive microenvironment. Cancer Cell. 2021;39:1464–78.e8. 34719426 10.1016/j.ccell.2021.09.005 PMC8578470

[117] Li XF, Selli C, Zhou HL, Cao J, Wu S, Ma RY, et al. Macrophages promote anti-androgen resistance in prostate cancer bone disease. J Exp Med. 2023;220:e20221007. 36749798 10.1084/jem.20221007 PMC9948761

[118] Jiao S, Subudhi SK, Aparicio A, Ge Z, Guan B, Miura Y, et al. Differences in tumor microenvironment dictate T helper lineage polarization and response to immune checkpoint therapy. Cell. 2019;179:1177–90.e13. 31730856 10.1016/j.cell.2019.10.029

[119] Beer TM, Kwon ED, Drake CG, Fizazi K, Logothetis C, Gravis G, et al. Randomized, double-blind, phase III trial of ipilimumab versus placebo in asymptomatic or minimally symptomatic patients with metastatic chemotherapy-naive castration-resistant prostate cancer. J Clin Oncol. 2017;35:40–7. 28034081 10.1200/JCO.2016.69.1584

[120] Landi L, D’Inca F, Gelibter A, Chiari R, Grossi F, Delmonte A, et al. Bone metastases and immunotherapy in patients with advanced non-small cell lung cancer. J Immunother Cancer. 2019;7:316. 31752994 10.1186/s40425-019-0793-8 PMC6868703

[121] Barnholtz-Sloan JS, Sloan AE, Davis FG, Vigneau FD, Lai P, Sawaya RE. Incidence proportions of brain metastases in patients diagnosed (1973 to 2001) in the Metropolitan Detroit Cancer Surveillance System. J Clin Oncol. 2004;22:2865–72. 15254054 10.1200/JCO.2004.12.149

[122] Quail DF, Joyce JA. The microenvironmental landscape of brain tumors. Cancer Cell. 2017;31:326–41. 28292436 10.1016/j.ccell.2017.02.009 PMC5424263

[123] Bowman RL, Klemm F, Akkari L, Pyonteck SM, Sevenich L, Quail DF, et al. Macrophage ontogeny underlies differences in tumor-specific education in brain malignancies. Cell Rep. 2016;17:2445–59. 27840052 10.1016/j.celrep.2016.10.052 PMC5450644

[124] Quail DF, Bowman RL, Akkari L, Quick ML, Schuhmacher AJ, Huse JT, et al. The tumor microenvironment underlies acquired resistance to CSF-1R inhibition in gliomas. Science. 2016;352:aad3018. 27199435 10.1126/science.aad3018 PMC5450629

[125] Qiao S, Qian Y, Xu G, Luo Q, Zhang Z. Long-term characterization of activated microglia/macrophages facilitating the development of experimental brain metastasis through intravital microscopic imaging. J Neuroinflammation. 2019;16:4. 30616691 10.1186/s12974-018-1389-9 PMC6323850

[126] Goldberg SB, Gettinger SN, Mahajan A, Chiang AC, Herbst RS, Sznol M, et al. Pembrolizumab for patients with melanoma or non-small cell lung cancer and untreated brain metastases: early analysis of a nonrandomized, open-label, phase 2 trial. Lancet Oncol. 2016;17:976–83. 27267608 10.1016/S1470-2045(16)30053-5 PMC5526047

[127] Klemm F, Mockl A, Salamero-Boix A, Alekseeva T, Schaffer A, Schulz M, et al. Compensatory CSF2-driven macrophage activation promotes adaptive resistance to CSF1R inhibition in breast-to-brain metastasis. Nat Cancer. 2021;2:1086–101. 35121879 10.1038/s43018-021-00254-0

[128] Leibold AT, Monaco GN, Dey M. The role of the immune system in brain metastasis. Curr Neurobiol. 2019;10:33–48. 31097897 PMC6513348

[129] Pukrop T, Dehghani F, Chuang HN, Lohaus R, Bayanga K, Heermann S, et al. Microglia promote colonization of brain tissue by breast cancer cells in a Wnt-dependent way. Glia. 2010;58:1477–89. 20549749 10.1002/glia.21022

[130] Karimi E, Yu MW, Maritan SM, Perus LJM, Rezanejad M, Sorin M, et al. Single-cell spatial immune landscapes of primary and metastatic brain tumors. Nature. 2023;614:555–63. 36725935 10.1038/s41586-022-05680-3 PMC9931580

[131] Whiteley AE, Ma D, Wang L, Yu SY, Yin C, Price TT, et al. Breast cancer exploits neural signaling pathways for bone-to-meninges metastasis. Science. 2024;384:eadh5548. 38900896 10.1126/science.adh5548 PMC12167639

[132] Gysler SM, Drapkin R. Tumor innervation: peripheral nerves take control of the tumor microenvironment. J Clin Investig. 2021;131:e147276. 10.1172/JCI147276 PMC8159682 34060481

[133] Reavis HD, Chen HI, Drapkin R. Tumor innervation: cancer has some nerve. Trends Cancer. 2020;6:1059–67. 32807693 10.1016/j.trecan.2020.07.005 PMC7688507

[134] Zahalka AH, Frenette PS. Nerves in cancer. Nat Rev Cancer. 2020;20:143–57. 31974491 10.1038/s41568-019-0237-2 PMC7709871

[135] Ayala GE, Dai H, Powell M, Li R, Ding Y, Wheeler TM, et al. Cancer-related axonogenesis and neurogenesis in prostate cancer. Clin Cancer Res. 2008;14:7593–603. 19047084 10.1158/1078-0432.CCR-08-1164

[136] Faulkner S, Jobling P, March B, Jiang CC, Hondermarck H. Tumor neurobiology and the war of nerves in cancer. Cancer Discov. 2019;9:702–10. 30944117 10.1158/2159-8290.CD-18-1398

[137] Amit M, Na’ara S, Gil Z. Mechanisms of cancer dissemination along nerves. Nat Rev Cancer. 2016;16:399–408. 27150016 10.1038/nrc.2016.38

[138] Cortese N, Rigamonti A, Mantovani A, Marchesi F. The neuro-immune axis in cancer: relevance of the peripheral nervous system to the disease. Immunol Lett. 2020;227:60–5. 32827634 10.1016/j.imlet.2020.07.010

[139] Ordovas-Montanes J, Rakoff-Nahoum S, Huang S, Riol-Blanco L, Barreiro O, von Andrian UH. The regulation of immunological processes by peripheral neurons in homeostasis and disease. Trends Immunol. 2015;36:578–604. 26431937 10.1016/j.it.2015.08.007 PMC4592743

[140] Cavel O, Shomron O, Shabtay A, Vital J, Trejo-Leider L, Weizman N, et al. Endoneurial macrophages induce perineural invasion of pancreatic cancer cells by secretion of GDNF and activation of RET tyrosine kinase receptor. Cancer Res. 2012;72:5733–43. 22971345 10.1158/0008-5472.CAN-12-0764

[141] Bakst RL, Xiong H, Chen CH, Deborde S, Lyubchik A, Zhou Y, et al. Inflammatory monocytes promote perineural invasion via CCL2-mediated recruitment and cathepsin B expression. Cancer Res. 2017;77:6400–14. 28951461 10.1158/0008-5472.CAN-17-1612 PMC5831809

[142] Renz BW, Tanaka T, Sunagawa M, Takahashi R, Jiang Z, Macchini M, et al. Cholinergic signaling via muscarinic receptors directly and indirectly suppresses pancreatic tumorigenesis and cancer stemness. Cancer Discov. 2018;8:1458–73. 30185628 10.1158/2159-8290.CD-18-0046 PMC6214763

[143] Di Chiaro P, Nacci L, Arco F, Brandini S, Polletti S, Palamidessi A, et al. Mapping functional to morphological variation reveals the basis of regional extracellular matrix subversion and nerve invasion in pancreatic cancer. Cancer Cell. 2024;42:662–81 e10. 38518775 10.1016/j.ccell.2024.02.017

[144] Marchesi F, Piemonti L, Fedele G, Destro A, Roncalli M, Albarello L, et al. The chemokine receptor CX3CR1 is involved in the neural tropism and malignant behavior of pancreatic ductal adenocarcinoma. Cancer Res. 2008;68:9060–9. 18974152 10.1158/0008-5472.CAN-08-1810

[145] Ceyhan GO, Demir IE, Rauch U, Bergmann F, Muller MW, Buchler MW, et al. Pancreatic neuropathy results in “neural remodeling” and altered pancreatic innervation in chronic pancreatitis and pancreatic cancer. Am J Gastroenterol. 2009;104:2555–65. 19568227 10.1038/ajg.2009.380

[146] Demir IE, Friess H, Ceyhan GO. Neural plasticity in pancreatitis and pancreatic cancer. Nat Rev Gastroenterol Hepatol. 2015;12:649–59. 26460352 10.1038/nrgastro.2015.166

[147] Ceyhan GO, Bergmann F, Kadihasanoglu M, Altintas B, Demir IE, Hinz U, et al. Pancreatic neuropathy and neuropathic pain–a comprehensive pathomorphological study of 546 cases. Gastroenterology. 2009;136:177–86 e1. 18992743 10.1053/j.gastro.2008.09.029

[148] Bapat AA, Hostetter G, Von Hoff DD, Han H. Perineural invasion and associated pain in pancreatic cancer. Nat Rev Cancer. 2011;11:695–707. 21941281 10.1038/nrc3131

[149] De Logu F, Marini M, Landini L, Souza Monteiro de Araujo D, Bartalucci N, Trevisan G, et al. Peripheral nerve resident macrophages and schwann cells mediate cancer-induced pain. Cancer Res. 2021;81:3387–401. 33771895 10.1158/0008-5472.CAN-20-3326 PMC8260461

[150] Tan DS, Agarwal R, Kaye SB. Mechanisms of transcoelomic metastasis in ovarian cancer. Lancet Oncol. 2006;7:925–34. 17081918 10.1016/S1470-2045(06)70939-1

[151] Clark R, Krishnan V, Schoof M, Rodriguez I, Theriault B, Chekmareva M, et al. Milky spots promote ovarian cancer metastatic colonization of peritoneal adipose in experimental models. Am J Pathol. 2013;183:576–91. 23885715 10.1016/j.ajpath.2013.04.023 PMC3730760

[152] Jonjic N, Peri G, Bernasconi S, Sciacca FL, Colotta F, Pelicci P, et al. Expression of adhesion molecules and chemotactic cytokines in cultured human mesothelial cells. J Exp Med. 1992;176:1165–74. 1383376 10.1084/jem.176.4.1165 PMC2119405

[153] Lanfrancone L, Boraschi D, Ghiara P, Falini B, Grignani F, Peri G. et al. Human peritoneal mesothelial cells produce many cytokines (granulocyte colony-stimulating factor [CSF], granulocyte-monocyte-CSF, macrophage-CSF, interleukin-1 [IL-1], and IL-6) and are activated and stimulated to grow by IL-1. Blood. 1992;80:2835–42. 1280480

[154] Mantovani A, Peri G, Polentarutti N, Bolis G, Mangioni C, Spreafico F. Effects on in vitro tumor growth of macrophages isolated from human ascitic ovarian tumors. Int J Cancer. 1979;23:157–64. 761940 10.1002/ijc.2910230204

[155] Mantovani A, Polentarutti N, Peri G, Shavit ZB, Vecchi A, Bolis G, et al. Cytotoxicity on tumor cells of peripheral blood monocytes and tumor-associated macrophages in patients with ascites ovarian tumors. J Natl Cancer Inst. 1980;64:1307–15. 6246299 10.1093/jnci/64.6.1307

[156] Mantovani A, Bottazzi B, Colotta F, Sozzani S, Ruco L. The origin and function of tumor-associated macrophages. Immunol Today. 1992;13:265–70. 1388654 10.1016/0167-5699(92)90008-U

[157] Peri G, Polentarutti N, Sessa C, Mangioni C, Mantovani A. Tumoricidal activity of macrophages isolated from human ascitic and solid ovarian carcinomas: augmentation by interferon, lymphokines and endotoxin. Int J Cancer. 1981;28:143–52. 6172388 10.1002/ijc.2910280206

[158] Bleriot C, Dunsmore G, Alonso-Curbelo D, Ginhoux F. A temporal perspective for tumor-associated macrophage identities and functions. Cancer Cell. 2024;42:747–58. 38670090 10.1016/j.ccell.2024.04.002

[159] Negus RP, Stamp GW, Relf MG, Burke F, Malik ST, Bernasconi S, et al. The detection and localization of monocyte chemoattractant protein-1 (MCP-1) in human ovarian cancer. J Clin Investig. 1995;95:2391–6. 7738202 10.1172/JCI117933 PMC295866

[160] Etzerodt A, Moulin M, Doktor TK, Delfini M, Mossadegh-Keller N, Bajenoff M, et al. Tissue-resident macrophages in omentum promote metastatic spread of ovarian cancer. J Exp Med. 2020;217:e20191869. 31951251 10.1084/jem.20191869 PMC7144521

[161] Ma X. The omentum, a niche for premetastatic ovarian cancer. J Exp Med. 2020;217:e20192312. 32103261 10.1084/jem.20192312 PMC7144536

[162] Bain CC, Hawley CA, Garner H, Scott CL, Schridde A, Steers NJ, et al. Long-lived self-renewing bone marrow-derived macrophages displace embryo-derived cells to inhabit adult serous cavities. Nat Commun. 2016;7:ncomms11852. 27292029 10.1038/ncomms11852 PMC4910019

[163] Cai DL, Jin LP. Immune cell population in ovarian tumor microenvironment. J Cancer. 2017;8:2915–23. 28928882 10.7150/jca.20314 PMC5604442

[164] Colvin EK. Tumor-associated macrophages contribute to tumor progression in ovarian cancer. Front Oncol. 2014;4:137. 24936477 10.3389/fonc.2014.00137 PMC4047518

[165] Nowak M, Klink M. The role of tumor-associated macrophages in the progression and chemoresistance of ovarian cancer. Cells. 2020;9:1299. 10.3390/cells9051299 PMC7290435 32456078

[166] Reinartz S, Schumann T, Finkernagel F, Wortmann A, Jansen JM, Meissner W, et al. Mixed-polarization phenotype of ascites-associated macrophages in human ovarian carcinoma: correlation of CD163 expression, cytokine levels and early relapse. Int J Cancer. 2014;134:32–42. 23784932 10.1002/ijc.28335 PMC4232932

[167] Song M, Yeku OO, Rafiq S, Purdon T, Dong X, Zhu L, et al. Tumor derived UBR5 promotes ovarian cancer growth and metastasis through inducing immunosuppressive macrophages. Nat Commun. 2020;11:6298. 33293516 10.1038/s41467-020-20140-0 PMC7722725

[168] Worzfeld T, Pogge von Strandmann E, Huber M, Adhikary T, Wagner U, Reinartz S, et al. The unique molecular and cellular microenvironment of ovarian cancer. Front Oncol. 2017;7:24. 28275576 10.3389/fonc.2017.00024 PMC5319992

[169] Xia H, Li S, Li X, Wang W, Bian Y, Wei S, et al. Autophagic adaptation to oxidative stress alters peritoneal residential macrophage survival and ovarian cancer metastasis. JCI Insight. 2020;5:e141115. 10.1172/jci.insight.141115 PMC7526547 32780724

[170] Yuan X, Zhang J, Li D, Mao Y, Mo F, Du W, et al. Prognostic significance of tumor-associated macrophages in ovarian cancer: a meta-analysis. Gynecol Oncol. 2017;147:181–7. 28698008 10.1016/j.ygyno.2017.07.007

[171] Hagemann T, Wilson J, Burke F, Kulbe H, Li NF, Pluddemann A, et al. Ovarian cancer cells polarize macrophages toward a tumor-associated phenotype. J Immunol. 2006;176:5023–32. 16585599 10.4049/jimmunol.176.8.5023

[172] Chen X, Ying X, Wang X, Wu X, Zhu Q, Wang X. Exosomes derived from hypoxic epithelial ovarian cancer deliver microRNA-940 to induce macrophage M2 polarization. Oncol Rep. 2017;38:522–8. 28586039 10.3892/or.2017.5697

[173] Ying X, Wu Q, Wu X, Zhu Q, Wang X, Jiang L, et al. Epithelial ovarian cancer-secreted exosomal miR-222-3p induces polarization of tumor-associated macrophages. Oncotarget. 2016;7:43076–87. 27172798 10.18632/oncotarget.9246 PMC5190009

[174] Allavena P, Chieppa M, Bianchi G, Solinas G, Fabbri M, Laskarin G, et al. Engagement of the mannose receptor by tumoral mucins activates an immune suppressive phenotype in human tumor-associated macrophages. Clin Dev Immunol. 2010;2010:547179. 21331365 10.1155/2010/547179 PMC3038419

[175] Neyen C, Pluddemann A, Mukhopadhyay S, Maniati E, Bossard M, Gordon S, et al. Macrophage scavenger receptor a promotes tumor progression in murine models of ovarian and pancreatic cancer. J Immunol. 2013;190:3798–805. 23447685 10.4049/jimmunol.1203194 PMC3608578

[176] Biswas SK, Mantovani A. Orchestration of metabolism by macrophages. Cell Metab. 2012;15:432–7. 22482726 10.1016/j.cmet.2011.11.013

[177] Geeraerts X, Bolli E, Fendt SM, Van Ginderachter JA. Macrophage metabolism as therapeutic target for cancer, atherosclerosis, and obesity. Front Immunol. 2017;8:289. 28360914 10.3389/fimmu.2017.00289 PMC5350105

[178] Zeitler L, Murray PJ. IL4i1 and IDO1: oxidases that control a tryptophan metabolic nexus in cancer. J Biol Chem. 2023;299:104827. 37196768 10.1016/j.jbc.2023.104827 PMC10318530

[179] Rodriguez GC, Haisley C, Hurteau J, Moser TL, Whitaker R, Bast RC Jr, et al. Regulation of invasion of epithelial ovarian cancer by transforming growth factor-beta. Gynecol Oncol. 2001;80:245–53. 11161867 10.1006/gyno.2000.6042

[180] Liu L, Wang X, Li X, Wu X, Tang M, Wang X. Upregulation of IGF1 by tumor-associated macrophages promotes the proliferation and migration of epithelial ovarian cancer cells. Oncol Rep. 2018;39:818–26. 29251331 10.3892/or.2017.6148

[181] Wang X, Zhu Q, Lin Y, Wu L, Wu X, Wang K, et al. Crosstalk between TEMs and endothelial cells modulates angiogenesis and metastasis via IGF1-IGF1R signaling in epithelial ovarian cancer. Br J Cancer. 2017;117:1371–82. 28898232 10.1038/bjc.2017.297 PMC5672923

[182] Kulbe H, Thompson R, Wilson JL, Robinson S, Hagemann T, Fatah R, et al. The inflammatory cytokine tumor necrosis factor-alpha generates an autocrine tumor-promoting network in epithelial ovarian cancer cells. Cancer Res. 2007;67:585–92. 17234767 10.1158/0008-5472.CAN-06-2941 PMC2679985

[183] Malik ST, Griffin DB, Fiers W, Balkwill FR. Paradoxical effects of tumor necrosis factor in experimental ovarian cancer. Int J Cancer. 1989;44:918–25. 2583871 10.1002/ijc.2910440529

[184] Hagemann T, Wilson J, Kulbe H, Li NF, Leinster DA, Charles K, et al. Macrophages induce invasiveness of epithelial cancer cells via NF-kappa B and JNK. J Immunol. 2005;175:1197–205. 16002723 10.4049/jimmunol.175.2.1197

[185] Kenny HA, Chiang CY, White EA, Schryver EM, Habis M, Romero IL, et al. Mesothelial cells promote early ovarian cancer metastasis through fibronectin secretion. J Clin Investig. 2014;124:4614–28. 25202979 10.1172/JCI74778 PMC4191043

[186] Carroll MJ, Fogg KC, Patel HA, Krause HB, Mancha AS, Patankar MS, et al. Alternatively, activated macrophages upregulate mesothelial expression of P-selectin to enhance adhesion of ovarian cancer cells. Cancer Res. 2018;78:3560–73. 29739756 10.1158/0008-5472.CAN-17-3341 PMC6030435

[187] Sodek KL, Ringuette MJ, Brown TJ. Compact spheroid formation by ovarian cancer cells is associated with contractile behavior and an invasive phenotype. Int J Cancer. 2009;124:2060–70. 19132753 10.1002/ijc.24188

[188] Yin M, Li X, Tan S, Zhou HJ, Ji W, Bellone S, et al. Tumor-associated macrophages drive spheroid formation during early transcoelomic metastasis of ovarian cancer. J Clin Investig. 2016;126:4157–73. 27721235 10.1172/JCI87252 PMC5096908

[189] Miyamoto T, Murphy B, Zhang N. Intraperitoneal metastasis of ovarian cancer: new insights on resident macrophages in the peritoneal cavity. Front Immunol. 2023;14:1104694. 37180125 10.3389/fimmu.2023.1104694 PMC10167029

[190] Kajiyama H, Shibata K, Terauchi M, Ino K, Nawa A, Kikkawa F. Involvement of SDF-1alpha/CXCR4 axis in the enhanced peritoneal metastasis of epithelial ovarian carcinoma. Int J Cancer. 2008;122:91–9. 17893878 10.1002/ijc.23083

[191] Lane D, Matte I, Laplante C, Garde-Granger P, Carignan A, Bessette P, et al. CCL18 from ascites promotes ovarian cancer cell migration through proline-rich tyrosine kinase 2 signaling. Mol Cancer. 2016;15:58. 27613122 10.1186/s12943-016-0542-2 PMC5017134

[192] Schutyser E, Struyf S, Proost P, Opdenakker G, Laureys G, Verhasselt B, et al. Identification of biologically active chemokine isoforms from ascitic fluid and elevated levels of CCL18/pulmonary and activation-regulated chemokine in ovarian carcinoma. J Biol Chem. 2002;277:24584–93. 11978786 10.1074/jbc.M112275200

[193] Asem M, Young AM, Oyama C, Claure De La Zerda A, Liu Y, Yang J, et al. Host Wnt5a potentiates microenvironmental regulation of ovarian cancer metastasis. Cancer Res. 2020;80:1156–70. 31932454 10.1158/0008-5472.CAN-19-1601 PMC8245162

[194] Allavena P, Introna M, Mangioni C, Mantovani A. Inhibition of natural killer activity by tumor-associated lymphoid cells from ascites ovarian carcinomas. J Natl Cancer Inst. 1981;67:319–25. 6943371

[195] Bellora F, Castriconi R, Dondero A, Pessino A, Nencioni A, Liggieri G, et al. TLR activation of tumor-associated macrophages from ovarian cancer patients triggers cytolytic activity of NK cells. Eur J Immunol. 2014;44:1814–22. 24510590 10.1002/eji.201344130

[196] Molgora M, Supino D, Mavilio D, Santoni A, Moretta L, Mantovani A, et al. The yin-yang of the interaction between myelomonocytic cells and NK cells. Scand J Immunol. 2018;88:e12705. 30048003 10.1111/sji.12705 PMC6485394

[197] Gottlieb CE, Mills AM, Cross JV, Ring KL. Tumor-associated macrophage expression of PD-L1 in implants of high grade serous ovarian carcinoma: A comparison of matched primary and metastatic tumors. Gynecol Oncol. 2017;144:607–12. 28065619 10.1016/j.ygyno.2016.12.021

[198] Kryczek I, Zou L, Rodriguez P, Zhu G, Wei S, Mottram P, et al. B7-H4 expression identifies a novel suppressive macrophage population in human ovarian carcinoma. J Exp Med. 2006;203:871–81. 16606666 10.1084/jem.20050930 PMC2118300

[199] Zhou J, Li X, Wu X, Zhang T, Zhu Q, Wang X, et al. Exosomes released from tumor-associated macrophages transfer miRNAs that induce a Treg/Th17 cell imbalance in epithelial ovarian cancer. Cancer Immunol Res. 2018;6:1578–92. 30396909 10.1158/2326-6066.CIR-17-0479

[200] Fetahu IS, Esser-Skala W, Dnyansagar R, Sindelar S, Rifatbegovic F, Bileck A, et al. Single-cell transcriptomics and epigenomics unravel the role of monocytes in neuroblastoma bone marrow metastasis. Nat Commun. 2023;14:3620. 37365178 10.1038/s41467-023-39210-0 PMC10293285

[201] LaMarche NM, Hegde S, Park MD, Maier BB, Troncoso L, Le Berichel J, et al. An IL-4 signaling axis in bone marrow drives pro-tumorigenic myelopoiesis. Nature. 2024;625:166–74. 38057662 10.1038/s41586-023-06797-9 PMC11189607

